# The landscape of enteric pathogen exposure of young children in public domains of low-income, urban Kenya: The influence of exposure pathway and spatial range of play on multi-pathogen exposure risks

**DOI:** 10.1371/journal.pntd.0007292

**Published:** 2019-03-27

**Authors:** Danielle Medgyesi, Daniel Sewell, Reid Senesac, Oliver Cumming, Jane Mumma, Kelly K. Baker

**Affiliations:** 1 Department of Occupational and Environmental Health, University of Iowa, Iowa City, Iowa, United States of America; 2 Department of Biostatistics, University of Iowa, Iowa City, Iowa, United States of America; 3 Department of Disease Control, London School of Hygiene and Tropical Medicine, London, United Kingdom; 4 Department of Community Nutrition, Great Lakes University of Kisumu, Kisumu, Kenya; Johns Hopkins Bloomberg School of Public Health, UNITED STATES

## Abstract

Young children are infected by a diverse variety of enteric pathogens in low-income, high-burden countries. Little is known about which conditions pose the greatest risk for enteric pathogen exposure and infection. Young children frequently play in residential public areas around their household, including areas contaminated by human and animal feces, suggesting these exposures are particularly hazardous. The objective of this study was to examine how the dose of six types of common enteric pathogens, and the probability of exposure to one or multiple enteric pathogens for young children playing at public play areas in Kisumu, Kenya is influenced by the type and frequency of child play behaviors that result in ingestion of soil or surface water. Additionally, we examine how pathogen doses and multi-pathogen exposure are modified by spatial variability in the number of public areas children are exposed to in their neighborhood. A Bayesian framework was employed to obtain the posterior distribution of pathogen doses for a certain number of contacts. First, a multivariate mixed effects tobit model was used to obtain the posterior distribution of pathogen concentrations, and their interdependencies, in soil and surface water, based upon empirical data of enteric pathogen contamination in three neighborhoods of Kisumu. Then, exposure doses were estimated using behavioral contact parameters from previous studies and contrasted under different exposure conditions. Pathogen presence and concentration in soil varied widely across local (< 25 meter radius area) and neighborhood-level scales, but pathogens were correlated among distinct surface water samples collected near to each other. Multi-pathogen exposure of children at public play areas was common. Pathogen doses and the probability of multi-pathogen ingestion increased with: higher frequency of environmental contact, especially for surface water; larger volume of soil or water ingested; and with play at multiple sites in the neighborhood versus single site play. Child contact with surface water and soil at public play areas in their neighborhood is an important cause of exposure to enteric pathogens in Kisumu, and behavioral, environmental, and spatial conditions are determinants of exposure.

## Author summary

The enteric infections that cause diarrheal disease remain a leading cause of morbidity and mortality in young children in low-income settings where sanitation infrastructure is limited. The role of different exposure pathways in transmission of enteric pathogens to young children is unknown, but accurate information on relative risks posed by different pathways could improve how well intervention strategies are prioritized. New evidence reveals that young children often play at public areas in their neighborhood, including areas used for human and animal feces disposal, potentially exposing them to heavily contaminated environmental fomites. We describe a novel exposure assessment approach that examines how the dose of six different enteric pathogens, and the probability of exposure to multiple pathogens is influenced by behavioral, environmental, and spatial conditions in public areas of three low-income, urban neighborhoods of Kisumu, Kenya. We found that increased frequency of contact with the environment and volume of material ingested, especially for surface water, and increased contact with different neighborhood locations elevates dose and probability of multiple pathogen exposure. Our study demonstrates that playing in public areas poses a risk to children and provides new insights on how pathogen exposures vary across transmission pathways.

## Introduction

Children living in low income countries with poor sanitary conditions experience an average of 4 to 8 diarrheal episodes per year between birth and 2 years of age, [1] demonstrating that they are chronically exposed to enteric pathogens beginning in the first year of life. Furthermore, recent studies have highlighted wide diversity in the microbial etiology of early childhood (<5 years of age) enteric infection in such settings, suggesting that they are exposed to a variety of pathogenic organisms in the first years of life.[2–9] Little is known about the rate with which children are exposed to and acquire enteric infections over time. While diarrheal incidence may suggest an exposure rate of up to 4 to 8 pathogen types per year, many infections are asymptomatic and go undetected without extensive diagnostic profiling, [10] meaning that diarrhea symptoms are likely an underestimate of how often children acquire new infections. Diarrhea rates may even further underestimate how often children are exposed to pathogens, but remain uninfected due to insufficient exposure dose, lack of pathogen viability, host acquired immunity, or other mediating conditions. Adding to this complexity, co-infection of individual children by two or more types of pathogens–regardless of symptomology–is common.[3, 11] Co-infection, even in the absence of diarrhea, is associated with greater risk of environmental enteric dysfunction (EED), undernutrition, and re-infection by a new pathogen, perpetuating the cycle of disease.[4] Understanding which exposure pathways contribute most to multi-pathogen exposure of children could improve the prioritization of interventions that reduce early childhood enteric disease incidence.

Wagner and Lanoix’s “F-diagram” conceptualized the routes of fecal-oral disease transmission according to the properties of environmental materials (drinking water, food, soil, etc.) that can be contaminated by feces and ingested by humans.[12] There is limited research on how exposure varies across exposure pathways, particularly with respect to the rates at which children experience multi-pathogen exposure and infection. Existing comparisons of exposure pathways have relied on fecal indicator bacteria concentrations, [13–18] or pathogen-specific risks, [19–21] both of which have major methodological limitations in measuring the overall probability of enteric pathogen exposure. Different types of pathogens have been frequently detected in households of India and Tanzania and public play areas in Kenya, revealing that exposure to pathogens in private and public settings is likely.[22–24] Our group has further shown that soil and surface water from public areas where children play in Kisumu are often contaminated *simultaneously* by multiple types of pathogens, [24] revealing that children ingesting soil or water at some public sites could ingest doses of multiple types pathogens. Only one report to our knowledge has examined exposure from the perspective of ingesting multiple types of pathogens, rather than presence/absence of an indicator.[25] But, the modeling approach summed the individual probabilities of exposure to each type of pathogen from South African surface waters, rather than accounting for interrelatedness of pathogen contamination across sampling locations or exposure pathways. Since multi-pathogen contamination varies across location, exposure models must account for possible pathway- or location-specific differences in multi-pathogen contamination and transference.

The overall probability of exposure to enteric pathogens may be fundamentally different across exposure pathways and across location of exposure. For example, eating soil from the ground, especially in public areas, may be more hazardous than ingestion of household drinking water because humans or animals may defecate directly on the ground whereas drinking water is more likely to be protected and treated for safety.[13, 26, 27] Young children typically have high rates of contact with soil and objects, [28–32] and occasionally surface water, [33, 34] and frequently place their hands in their mouth with no handwashing in between.[34, 35] This results in frequent indirect ingestion of trace amounts of soil, and perhaps surface water. Geophagia (direct ingestion of handfuls of soil) among young children [26, 27, 34, 36] and drinking from surface water also occur, albeit less frequently than hand-to-mouth behaviors.[27, 34, 36, 37] The relative contributions of different behaviors, volume of material ingested, and type of material on cumulative pathogen doses (total number of pathogen types ingested per day) is unknown.

Finally, spatial variability of young children’s play in neighborhoods could influence the dose and diversity of pathogen exposure. Many children play outside the household unattended, while others have developmental- or guardian- driven limitations that restrict distance away from the household and acceptable areas for play. In settings where the landscape is often contaminated by feces from many humans and animals, the children who play in a constrained spatial area (i.e. near their household) may have a lower probability of pathogen exposure than children who roam across a larger spatial area and play at a variety of locations throughout the course of a day, especially if those locations are public areas used for feces disposal. More knowledge on how child behavior, type of environmental fomite, and spatial range of child play influences enteric pathogen exposure is needed for prioritizing interventions.

This novel exposure assessment study utilizes information on fine and macro-scale spatial variability in enteric pathogen detection and co-detection across public play areas in a typical low-income, fecal-contaminated setting to explore the relative importance of different environmental, behavioral, and spatial conditions in pathogen exposure of young children. The first objective of this study was to measure how increased frequency of child contact with soil or surface water and the volume ingested (indirect vs. direct ingestion) by children in public play areas influences the ingestion dose of enteric pathogens, and the probability of exposure to one or more enteric pathogens. Second, we compare how site-constrained (child plays at one public residential location) versus neighborhood (free roaming) spatial range of play for children influences pathogen dose and probability of multi-pathogen exposure. This modeling approach could be adapted to include a variety of setting-specific information on child behaviors and environmental conditions to better assess the relative contribution of various exposure pathways to child infection.

## Materials and methods

### Study design

This exposure assessment study utilizes observational and environmental microbiology data on public sites in Kisumu, Kenya that has been described previously.[24] In brief, 166 total public sites in three peri-urban neighborhoods of Kisumu were randomly selected for a cross-sectional study on the role of human and animal sanitary conditions in neighborhood-level pathogen contamination. A “site” was defined as all public area (private households and businesses excluded) falling within a 25-meter radius of a randomly-generated set of central coordinates within each neighborhood. During rapid observation of sites (~10–15 minutes per site), our prior study revealed that at least one child <5 yrs was observed at 40% (66 of 166) of public sites, [24] with 94% (62 of 66 child observations) of these occurring in residential versus industrial, farming, or undeveloped areas. Furthermore, children were observed performing behaviors that resulted in hand or mouth contact with environmental fomites (touching soil, surface water, animals, or objects on the ground, swimming, eating food, eating dirt, mouthing hands). Soil and surface water samples analyzed by qRT-PCR were found to contain multiple types of common enteric pathogens.

This study uses microbial data reported in the parent study, but restricted to 116 residential public sites where children are most likely to spend time. In the parent study, three extra samples were collected at seven randomly-selected sites in each neighborhood to account for anticipated variance in pathogen distributions within the <25 m radius area of individual site. Resamples could have been as much as 50 meters apart, if on opposite ends of the 25 m radius site. A total of 125 soil samples and 34 surface water samples were collected from 116 residential public sites included in the study, including 15 sites where 45 soil and 3 sites where 6 surface waters were sampled.[24]

To ensure sufficient knowledge about the distribution of pathogen concentrations and to obtain numerical stability in the statistical estimation algorithms, only pathogens detected in greater than 5% of both soil and surface water samples from eligible sites were included in the model. Of the 19 pathogens tested during environmental sampling in the parent study, concentration data for 6 pathogens (S1 Table) were eligible for inclusion in the model: *Cryptosporidium spp*., *Giardia lamblia*, human adenovirus 40/41, Enteropathogenic *E*. *coli* (EPEC *bfpA* and/or *eaeA*), Enterotoxigenic *E*. *coli* (ETEC *estA* and/or *eltB*), and Enteroaggregative *E*. *coli* (EAEC *aaicA* and/or *aatA*).[24] Detection frequencies for less common pathogens at these 116 residential public sites are reported in S2 Table. If there was a positive detect for more than one bacterial gene marker, and concentrations (C_p_) varied between gene markers, concentrations of ETEC-*estA*, EPEC-*bfpA*, and EAEC-*aatA* were prioritized over concentrations of ETEC-*eltB*, EPEC-eaeA, and EAEC-*aaiC*, respectively, based on etiological importance in pediatric diarrheal disease.[2, 10] Although the dataset was restricted to 6 of 19 pathogens measured, the number of soil and surface water samples with at least one positive detect (138/159 samples) did not change, indicating that these 6 pathogens collectively are sensitive indicators for the presence of pathogen contamination in the environment in this setting. Repeat detection of each pathogen in <25 m radius multi-sampled sites is reported in S3 Table.

### Statistical analyses

The statistical analyses aimed to estimate the dose distributions of each pathogen type by environmental fomite type (soil vs. surface water), by contact type (indirect hand-to-mouth vs. direct geophagy or drinking), frequency of contact, and spatial range of exposure (site-restricted contact limited to <25 meter radius public environment vs. neighborhood-level contact with multiple randomly selected sites across the neighborhood). We also aimed to characterize fine scale within-site variability in pathogen contamination by sample type to understand how our sampling design might influence between-sample pathogen covariance. All analyses were conducted using R version 3.5.0. A Bayesian framework was employed to obtain the posterior distribution of pathogen doses for a certain number of contacts, denoted as *D*(*k*) for *k* contact events, i.e., the distribution of *D*(*k*) implied by information provided by both our data and previous studies.[15, 27, 32, 38–41] The posterior distribution yields point estimates and credible intervals for the parameters of the pathogen concentration distribution, denoted as *θ* and described in more detail below, for soil and water samples.

There are two parts to the modeling framework. The first part uses environmental microbiology data (S1 Table) to estimate the distribution of each pathogen concentration in soil and surface water.[24] The second part combines the concentration distribution of part one with contact fate parameters provided from previous studies (Table 1) to estimate the exposure pathway-specific dose distribution by fomite type, contact type, and behavior frequency. The posterior distribution of interest, namely that of *D*(*k*) and *θ* given the observed data and information from previous studies, can be decomposed to clearly reveal these two components of the statistical model:

Pr(D(k),θ|data,previousstudies)=Pr(D(k)|θ,previousstudies)⋅Pr(θ|data)

In implementation, we estimated this posterior distribution via a Monte Carlo approach on the joint posterior distribution augmented with the pathogen concentration corresponding to the *k* events. That is, we may first obtain a sample of *θ* from the marginal posterior distribution given the data, then draw *k* pathogen concentrations for the current value of *θ*, and finally, given those concentrations and information obtained from previous studies on exposure pathways, draw *D*(*k*).

**Table 1:** Description of variables and parameters used to estimate distribution of indirect and direct doses.

Description	Symbol	Parameter	Source
Pathogen concentration	C		
Soil/gram	^a^ C_Sp_	See S1 Table	This study
Surface water/mL	C_Wp_	See S1 Table	This study
Transfer efficiency from object	TE		
Soil adherence	TE_S_	Lognormal (0.52,0.9) mg/cm^2^	Finley et al. (1994) [39]
Water film thickness	TE_W_	Uniform (0.00234, 0.00499) cm	EPA Exposure (1987) [38]
Surface area of hand	SA_Hi_		
6 to <12 months	SA_H1_	Normal (240, 18.2) cm^2^	EPA Exposure (2011) [32]
12 to <24 months	SA_H2_	Normal (300, 30.4) cm^2^	EPA Exposure (2011) [32]
24 to <72 months ^b^	SA_H3_	Normal (347.5, 48.6) cm^2^	EPA Exposure (2011) [32]
Fraction of hand	F_H_		
Contacting object	F_HO_	Normal (0.215, 0.111) %	Auyueng et al. (2008) [40]
Contacting mouth	F_HM_	Normal (0.18, 0.076) %	Auyueng et al. (2008) [40]
Transfer efficiency from hand	TE_H_		
Hand-to-mouth	TE_HM_	0.33 (%)	Rusin et al. (2002) [41]
Volume ingested during direct ingestion	V		
Geophagia	V_S_	1.25 grams	Ngure et al. 2013 [27]
Drinking surface water	V_W_	5 mL	Labite et al. 2010 [15]

^a^ Converted C_Sp_ to mg

^b^ Averaged from reported data for age groups 2 to 3 years and 3 to 6 years.

### Distribution of pathogen concentrations in soil and surface water

Several challenges arose in estimating the parameters *θ* for the pathogen concentration distributions. First, there was left censoring caused by methodologically-constrained lower limits of detection (S1 Table). Second, there were two important sources of dependency in the data—that which occurs due to the correlations between the different pathogens, and that which occurs due to re-sampling within the 25-meter radius area of an individual site.

To handle data challenges, we fit a multivariate mixed effects (MVME) tobit model to the log transformed concentration data. The first source of dependency in the data was accounted for by modeling all pathogens jointly rather than running many univariate analyses. It was also important that we not neglect to account for the latter type of dependency described above, as the spatial patterns of young children playing in neighborhoods could influence the dose and diversity of pathogen exposure in public areas. Thus, the proposed random effects included in our MVME tobit model account for this spatial dependence. The parameters of the MVME tobit model *θ* can thus be broken into three components: (1) the mean of the log concentrations for each of the 6 pathogens; (2) the 6 × 6 covariance matrix for the residuals (Σ); (3) and the 6 × 6 covariance matrix of the random effects (Ω). Hence *θ* contains 48 parameters. The correlation for the *p*^th^ pathogen between two samples at the same site; equivalently, the proportion of total variance explained by the site-level variance can be found via the intra-class correlation (ICC): Ω_*pp*_/(Ω_*pp*_ + Σ_*pp*_), and the total variance of the *p*^th^ pathogen log concentration is Ω_*pp*_ + Σ_*pp*_. Of course, samples taken at two different sites are independent and is representative of our neighborhood-level play scenario. See Supporting Information S1 Text for details on this statistical model.

For each environmental sample type, samples of *θ* were obtained from the posterior distribution using a Gibbs sampler. From these samples, posterior draws of pathogen concentrations of *k* new events were drawn from a multivariate normal distribution parameterized by the draws of *θ*. See Supporting Information S1 Text for details on the Gibbs sampling algorithm.

### Exposure pathway-specific dose distribution

A theoretical model was developed to estimate and compare the dose and diversity of enteric pathogens ingested by young children via indirect and direct exposure to soil and surface water at public play areas. The contact frequency was held at a constant rate, ranging from a minimum of 1 to a maximum of 10 contacts, for pattern comparison purposes, so behaviors in this model are not weighted to account for the likelihood of engaging in the behavior and the rate of contact given a child plays in a public area for a specified time span.[34] Therefore, the results are not cumulative estimates of actual child exposure, but represent possible exposures given a range of possible conditions. To obtain a posterior sample of the final dose for each set of conditions, the *k* concentrations drawn previously were multiplied by fate parameters, each drawn from a random distribution to account for the inherent variability in such occurrences, and then the *k* doses ranging from 1 to 10 were summed. The spatial assumption determined whether or not the *k* contacts were correlated. The formulas used to estimate the dose distribution from indirect and direct contact with soil (1) and surface water (2) are:

1. **Soil**
  $$\text{Soil}-{\text{Hand}-\text{Mouth}\;\text{Dose}}_{\text{p}}={\text{C}}_{\text{Sp}}\times {\text{TE}}_{\text{S}}\times {\text{SA}}_{\text{Hi}}\times *{\text{F}}_{\text{HO}}\times \left(†\frac{{*\text{F}}_{\text{HM}}}{{\text{F}}_{\text{HO}}}\right)\times {\text{TE}}_{\text{HM}}$$
  $${\text{Geophagia}\;\text{Dose}}_{\text{p}}{=\text{C}}_{\text{Sp}}{\times \text{V}}_{\text{S}}$$
2. **Surface water**
  $$\text{Water}-\text{Hand}-{\text{Mouth}\;\text{Dose}}_{\text{p}}={\text{C}}_{\text{Wp}}\times {\text{TE}}_{\text{W}}\times {\text{SA}}_{\text{Hi}}\times *{\text{F}}_{\text{HO}}\times \left(†\frac{*{\text{F}}_{\text{HM}}}{{\text{F}}_{\text{HO}}}\right)\times {\text{TE}}_{\text{HM}}$$
  $${\text{Drink}\;\text{Surface}\;\text{Water}\;\text{Dose}}_{\text{p}}={\text{C}}_{\text{Wp}}\times {\text{V}}_{\text{W}}$$

The *p*^*th*^ pathogen is denoted by a subscript of *p* and their concentrations in soil and surface water are denoted as C_Sp_ and C_Wp_, respectively. * means truncated with a lower bound = 0 and upper bound = 1, † means the surface area of hand-to-mouth contact cannot exceed the surface area that was contaminated during hand-to-object contact, thus the fraction is truncated at 1.

Fate parameters obtained from the extant literature to estimate exposure to pathogens through indirect contact include: the transfer efficiency of the environmental fomite to the hand (soil: TE_S_, water: TE_W_), the total surface area of the child’s hand (SA_Hi_), the fraction of the child’s hand contacting the environmental object (F_HO_), the fraction of the hand mouthed by the child (F_HM_), and the transfer efficiency of environmental residual from hand-to-mouth (TE_HM_) (Table 1). Total hand surface area (cm^2^) used in this model was based on estimated surface area parameters for children between the ages of six months to less than six years.[32] Standard deviation for total hand surface area (cm^2^) per age category (6 to 11 months, 12 to 23 months, and 24 to 72 months) was calculated by dividing the difference of the EPA-reported mean and 95^th^ percentile for hand surface area by the 95^th^ quantile of a standard normal distribution (1.645).[32] Each age category was equally represented during simulation by sampling the probability of obtaining a random child within each of the unequal month spans and respective hand surface area mean and standard deviation (SA_Hi_). The distribution for the fraction of the child’s hand involved in hand-to-object contact (F_HO_) and hand-to-mouth contact (F_HM_) was calculated by minimizing the squared differences between theoretical and empirical quantiles.[40] Transfer efficiency of environmental residue from hand-to-mouth (TE_HM_) was estimated with a single point estimate due to the lack of literature to infer a distribution for all pathogens used in this analysis.[41] Our exposure model assumes that the hand region that contacted the object is the same region that contacted the mouth. This assumption is supported by the finding that regardless of the type of interaction, hand contact predominantly involves the fingers.[40] When summing across estimated indirect doses, hand size was held constant, while soil adherence and hand area that contacted the object and then mouth varied between *k* contact events. To estimate direct ingestion of soil or surface water, the volume of respective substance placed in the mouth during a geophagia (V_S_) or drinking occurrence (V_W_) was estimated with a single parameter because of the lack of literature to describe the distribution of direct ingestion occurrences (Table 1).[15, 27, 33]

## Results

### Correlation in pathogen concentrations

We ran 25,000 iterations of the Gibbs sampler thinning by keeping every 5th draw. Of these 5,000 draws, 1,500 were used as a burn in period leaving 3,500 samples for our Monte Carlo analyses. The estimated pathogen concentration (*C*) log means and back-transformed median concentrations (exponentiated log mean) are reported in Table 2. The log standard deviation (SD) size relative to the log mean was large for pathogens in soil and surface water, except for the frequently detected *Cryptosporidium spp*. Intraclass correlation (ICC), indicating the correlation between pathogens in different samples at the same site, varied by pathogen and type of environmental material. Moderate-to-high ICCs in surface water indicated low within-site variability in concentration for all pathogens. Low-to-moderate ICC in soil indicated pathogen concentrations varied more within site.

**Table 2:** Tobit model-simulated log mean (95% credible intervals), median concentrations (conc.), total log standard deviation (SD) (95% credible intervals), and interclass correlation coefficient (ICC) of enteric viruses, bacteria, and protozoans detected per gram (g) of soil and milliliter (mL) of surface water public areas of Kisumu where children play.

Pathogen type	Soil per gram			
	Log mean (95% CI)	Median conc.	Log SD (95% CI)	^d^ ICC
*Cryptosporidium spp*.	11.38 (10.7, 12.05)	8.79x10^4^	3.47 (2.98, 4.05)	0.19
*Giardia lamblia*	0.08 (-4.68, 3.01)	1.09x10^0^	8.21 (6.35, 11.45)	0.39
human adenovirus 40/41	-0.07 (-7.74, 4.32)	9.30x10^-1^	9.27 (6.76, 13.98)	0.41
^a^ ETEC estA*/eltB	1.07 (-5.09, 4.35)	2.91x10^0^	8.26 (5.83, 12.97)	0.19
^b^ EPEC bfpA*/eaeA	-3.14 (-16.10, 2.92)	4.00x10^-2^	9.68 (5.90, 18.81)	0.56
^c^ EAEC aatA*/aaiC	-1.07 (-6.04, 2.64)	3.40x10^-1^	8.05 (5.66, 11.50)	0.37
	**Surface water per millilitre**			
*Cryptosporidium spp*.	7.57 (6.09, 9.01)	1.93x10^3^	4.06 (3.16, 5.29)	0.48
*Giardia lamblia*	1.95 (-10.05, 5.54)	7.04x10^0^	7.01 (3.34, 23.1)	0.77
human adenovirus 40/41	4.46 (-1.04, 8.50)	8.61x10^1^	8.97 (5.93, 14.06)	0.66
^a^ ETEC estA*/eltB	6.00 (4.41, 7.39)	4.04x10^2^	3.76 (2.80, 5.06)	0.65
^b^ EPEC bfpA*/eaeA	5.74 (3.28, 7.79)	3.10x10^2^	5.36 (3.78, 7.87)	0.68
^c^ EAEC aatA*/aaiC	6.32 (4.44, 7.98)	5.57x10^2^	4.45 (3.26, 6.30)	0.68

^a^ Enterotoxigenic *E*. *coli* (ETEC)

^b^ Enteropathogenic *E*. *coli* (EPEC)

^c^ Enteroaggregative *E*. *coli* (EAEC)

^d^ Intraclass correlation coefficient (ICC): proportion of total variance explained by the site-level variance; equivalently, the correlation between different samples taken at the same site.

**Fig 1:**
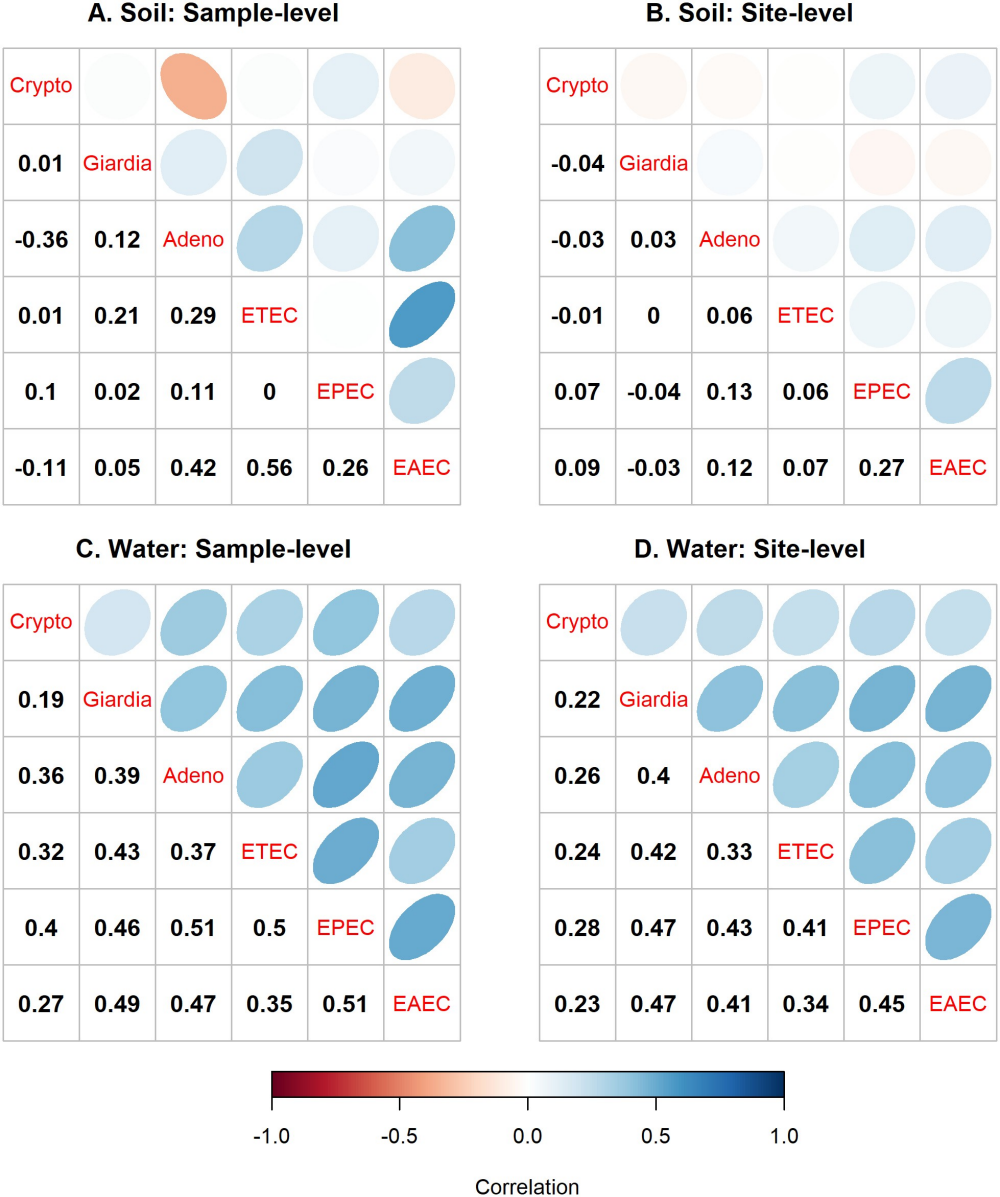
Sample- and site-level correlation in concentration of enteric pathogens in soil and surface water at public areas of Kisumu, Kenya. (A) Correlation in pathogen concentration in soil between all samples. (B) Correlation in pathogen concentrations in soil between multiple samples within a site (<25 meter radius area). (C) Correlation in pathogen concentration in surface water between all samples. (D) Correlation in pathogen concentrations in surface water among multiple samples within a site (<25 meter radius area). Negative and positive correlation shown in the lower left quadrants of each grid are reflected in orange-red and blue circles, respectively, in the upper right quadrants of each grid. The highest rho indicated by the darkest color and narrowest shapes.

Estimated pathogen concentration (*C*) distributions (Table 2) were all positively correlated in surface water, but many were not positively correlated in soil (Fig 1). The 95% credible intervals (CI) around the correlation means (S1 Fig) revealed that within a single sample of surface water, twelve of fifteen pathogen comparisons were significantly positively correlated. When comparing different water samples taken at the same site, six pathogen comparisons in surface water were significantly positively correlated: adenovirus 40/41 and EPEC, adenovirus 40/41 and EAEC, ETEC and EPEC, Giardia and EPEC, Giardia and EAEC, and EPEC and EAEC. Many other pathogen comparisons trended towards statistical significance. Within a single sample of soil, we observed a significant negative correlation between *Cryptosporidium spp*. and adenovirus 40/41 and positive correlations for ETEC and adenovirus, EAEC and adenovirus 40/41, and EAEC and ETEC. However, significant pathogen correlations were not observed between different samples from the same site, reflecting high variability in concentrations within a 25m radius area.

### Pathogen exposure doses by behavioral pathway and spatial range

**Fig 2:**
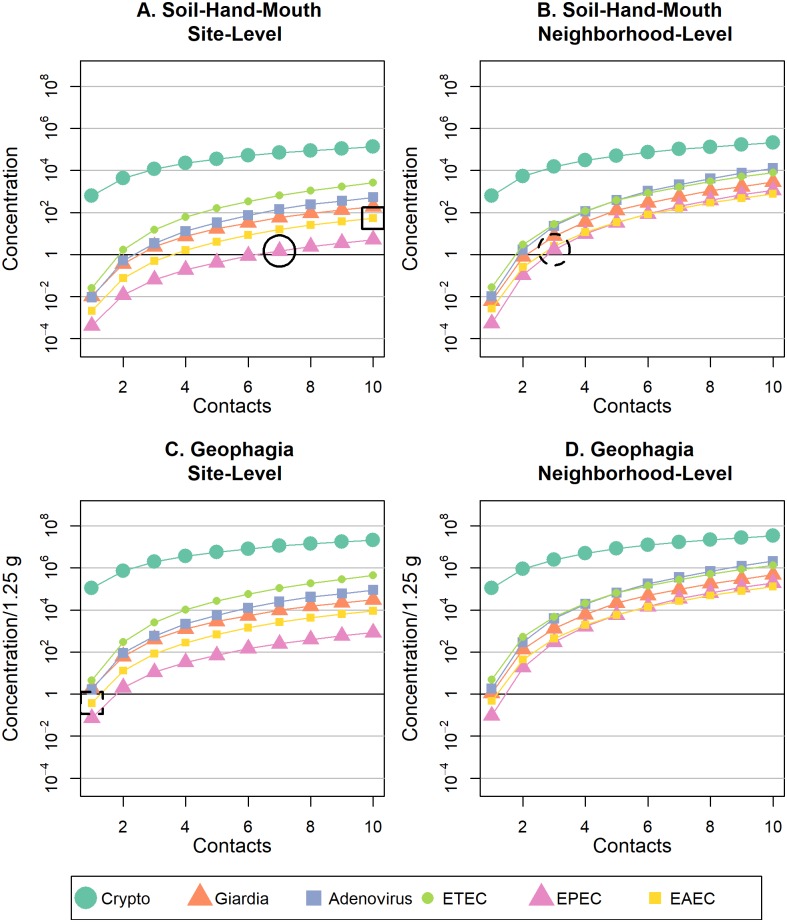
Mean dose of six enteric pathogens ingested with increased frequency of soil-hand-mouth or geophagy behaviors at site-restricted versus neighborhood levels of spatial scale. Mean pathogen doses are shown for (A) soil-hand-mouth exposure behaviors at a single residential public site; (B) soil-hand-mouth exposure behaviors at two or more residential public sites within a neighborhood; (C) geophagy behaviors at a single residential public site; and (D) geophagy behaviors at two or more residential public sites within a neighborhood.

**Fig 3:**
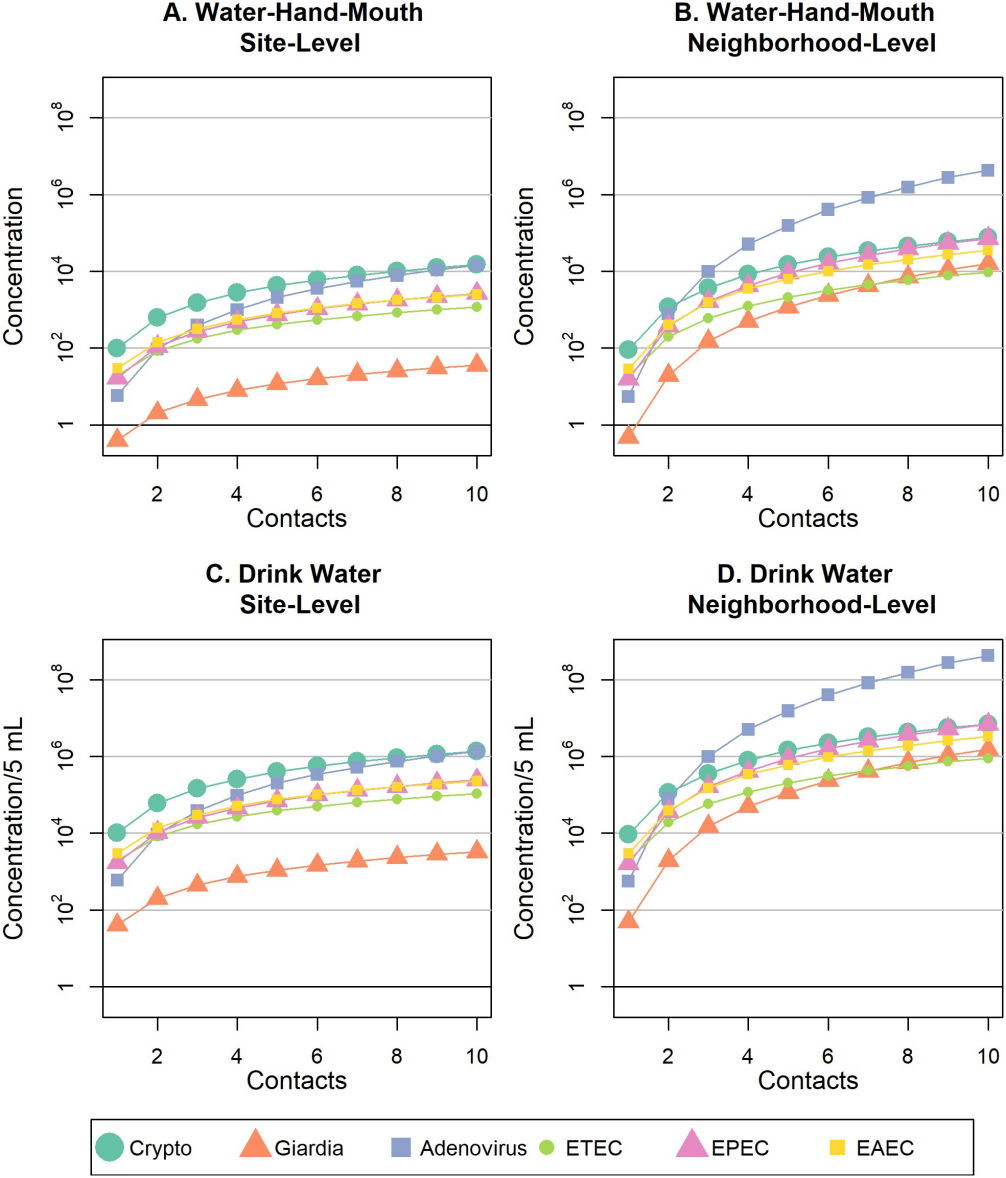
Mean dose of six enteric pathogens ingested with increased frequency of hand-to-mouth or surface water drinking behaviors at site-restricted versus neighborhood levels of spatial scale. Mean pathogen doses are shown for (A) water-hand-mouth exposure behaviors at a single residential public site; (B) water-hand-mouth exposure behaviors at two or more residential public sites within a neighborhood; (C) drinking surface water behaviors at a single residential public site; (D) drinking surface water behaviors at two or more residential public sites within a neighborhood.

When comparing similar pathway, number of contacts, and spatial scale, adenovirus, EPEC, and EAEC pathogen doses from soil contact (Fig 2) were always lower than doses from surface water (Fig 3), and Cryptosporidium doses were consistently higher from soil contact than surface water (see S2–S5 Figs for pathogen-specific graphs). As the rate of hand-to-mouth contact or direct ingestion increased for site-level play, the dose of Giardia and ETEC from soil exceeded that of surface water (S2 and S4 Figs). All contact with surface water resulted in ingestion of DNA representing at least one pathogen organism of any type, with the exception of Giardia, for one water-hand-mouth contact (Fig 3). If frequency of contact with soil or surface water is held constant, geophagia or drinking surface water always resulted in higher pathogen doses compared to soil/water-hand-mouth contact (Fig 2C/2D vs. 2A/2B; Fig 3C/3D vs. 3A/3B). However, if hand-mouth contact occurs more often than geophagy or drinking surface water, then doses resulting from hand-mouth contact could exceed exposures from direct ingestion. For example, if a child exhibited ten cumulative soil-hand-mouth contacts and one geophagia contact during play at one site, the EAEC dose for soil-hand-mouth (~53 bacteria, Fig 2A, solid box) would exceed the EAEC dose for geophagia (~0.4 bacteria, Fig 2C, dashed box). Overall, when frequency of contact with soil or surface water is held constant, pathogen doses were always greater when children played at multiple sites in the neighborhood, versus just one site (Fig 2B/2D vs. 2A/2C; Fig 3B/3D vs. 3A/3C), but the magnitude of change depended upon the pathogen type (visualized side-by-side in S6–S11 Figs).

Pathogen-specific dose distributions from indirect and direct contact with soil and surface water at site- and neighborhood-level are reported in S6–S11 Figs. The log mean and untransformed median dose *D*(*k*) of pathogens ingested during contact with soil were largest for *Cryptosporidium spp*. (Fig 2). However, *Cryptosporidium spp*. dose did not considerably increase with increased behavior frequency or increased spatial scale of play from site- to neighborhood-level (S6 Fig). The lowest pathogen dose ingested during soil contact was EPEC—yet the dose of EPEC substantially increased for neighborhood vs. site-level play (S10 Fig). For example, it required over two times more soil-hand-mouth contacts to ingest the same dose of EPEC at site-level play (~7 contacts, Fig 2A, solid circle) as neighborhood-level play (~3 contacts, Fig 2B, dashed circle). Noticeably, the dose of human adenovirus 40/41 from surface water contact exponentially increased as spatial scale expanded from site to neighborhood play and surpassed all other pathogen doses at neighborhood-level exposure.

Pathogen dose and multi-pathogen exposure could differ with age due to differences in frequency of child behaviors and child hand size. In this analysis, behavior frequency was treated either as an experimental factor influencing dose or a constant for comparing doses between soil versus water exposure pathways or site versus neighborhood exposure pathways. However, child hand size was a parameter in our model that increases with age and could influence soil-hand-mouth or water-hand-mouth pathogen transmission. A sensitivity analysis for age group (S12–S15 Figs and S4–S7 Tables) revealed only small differences in pathogen doses for children 6 to <12 month, 12 to <24 month, and 24 to <72 months of age, although older children who had slightly greater doses because of larger hand size.

### Exposure to diverse pathogen types

**Fig 4:**
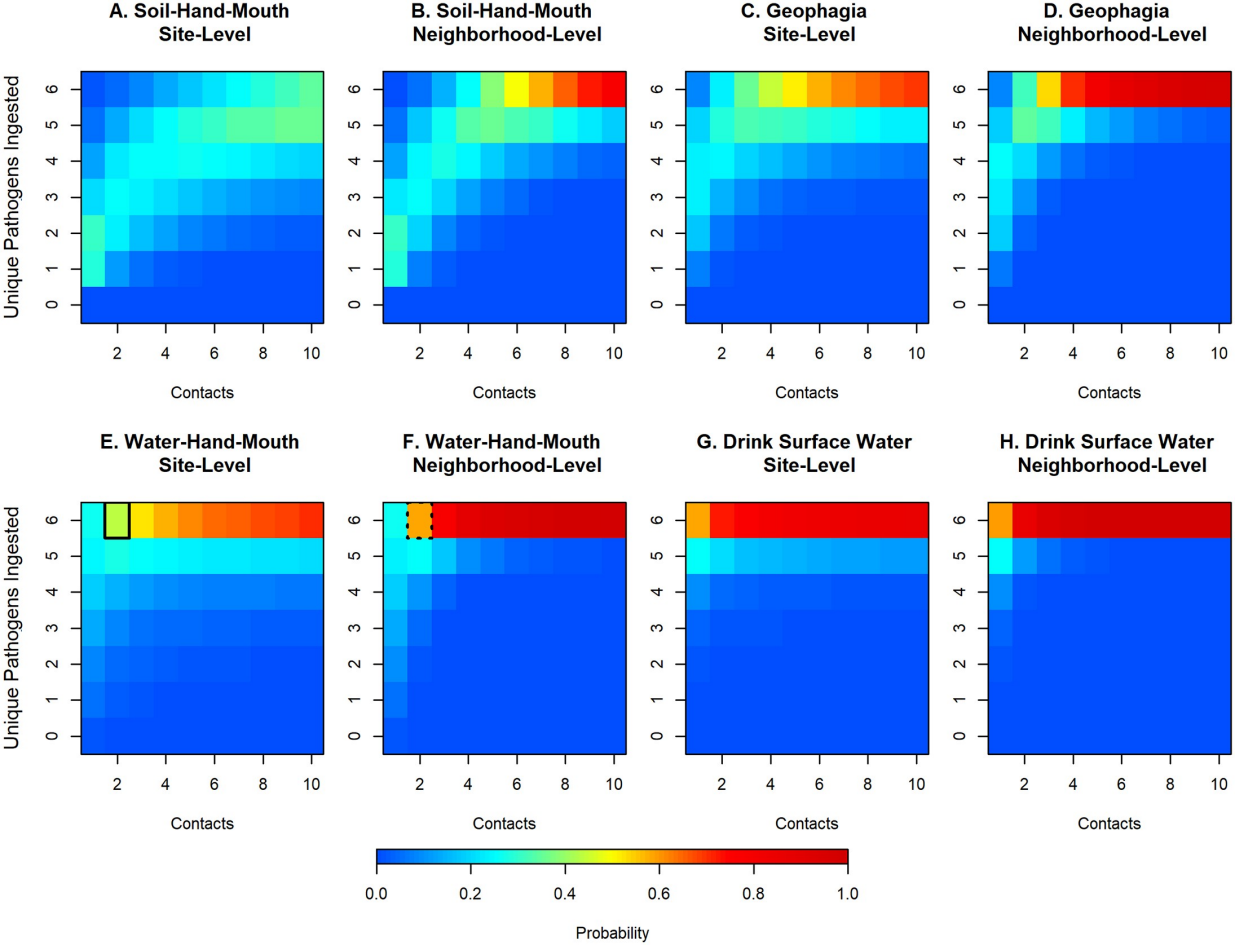
Probability of ingesting one or more enteric pathogens with increased frequency of exposure contact with soil or surface water at site-restricted and neighborhood levels of scale. Multi-pathogen exposure is shown for (A) soil-hand-mouth exposure behaviors at a single residential public site; (B) soil-hand-mouth exposure behaviors at two or more residential public sites within a neighborhood; (C) geophagy behaviors at a single residential public site. (D) geophagy behaviors at two or more residential public sites within a neighborhood; (E) water-hand-mouth exposure behaviors at a single residential public site; (F) water-hand-mouth exposure behaviors at two or more residential public sites within a neighborhood; (G) drinking surface water at a single residential public site; (H) drinking surface water at two or more residential public sites within a neighborhood.

Fig 4 illustrates the probability of ingesting one or more pathogens for 1 to 10 indirect or direct contact(s) with soil or surface water during site-restricted or neighborhood play, where a successful ingestion is defined as DNA of 1 or more pathogen types. Across all behaviors, the probability of ingesting more than one pathogen type intensified as spatial scale expanded from site to neighborhood play. For example, the probability of ingesting six pathogens from two water-hand-mouth contacts during site-restricted play (43%, Fig 4E, solid box) increased by about 15% if the child exhibited the same behavior and frequency during neighborhood-level play (58%, Fig 4F, dashed box). Soil-hand-mouth contact resulted in the lowest probability of ingesting diverse pathogen types compared to all other behaviors practiced at the same frequency. This is especially evident for soil-hand-mouth contact during site-restricted play where the probability of ingesting all 6 pathogens did not exceed 35% for 10 contacts. Any contact with surface water posed a high probability for ingestion of diverse pathogens and is demonstrated by a > 90% probability of ingesting 6 pathogens during ≥5 water-hand-mouth contacts and ≥3 drinking water contacts during neighborhood-level play.

## Discussion

Our prior work has demonstrated that children < 5 yrs living in low-income neighborhoods of Kisumu, Kenya are exposed during play in public residential areas to soil and surface water contaminated by human and animal feces and a diverse range of enteric pathogens.[24] This study addresses the next question as to how children are impacted by playing in these settings. Specifically, we quantified the impact of different child exposure behaviors, environmental transmission pathways, and spatial situations on the dose and diversity of enteric pathogens ingested by young children as a result of contact with public residential areas. When holding frequency of behaviors constant, the dose and probability of multiple enteric pathogen exposure were typically greater when a child ingested: (1) surface water versus soil, (2) greater volumes of soil (geophagy) or surface water (drinking small mouthfuls) versus soil/water-hand-mouth contact, and (3) soil or surface water from multiple neighborhood locations versus just one spatially-restricted site. Evidence that children have an increased probability of simultaneous exposure to multiple enteric pathogens during certain play conditions indicates that exposure pathways may be more important than others in elevating the risk of infection or even co-infection by different pathogens.

Our studies in Kenya and Haiti have confirmed that children engage in the exposure behaviors chosen for this exposure assessment (hand contact with soil and surface water, hand-to-mouth contact, and geophagy) in public areas and is consistent with extant literature in domestic settings. [26–28, 33, 34, 36, 42] In our studies of child play in public areas in Haiti, geophagy was 6 times more common than drinking surface water (0.9/hr vs 0.15/hr), and hand-to-mouth was roughly 9 (child) to 20 (infant) times more common than geophagy.[34] If we assume that child behavior frequencies are generalizable across geographic contexts, then child exposure to low doses (10^0^−10^2^) of at least one type of pathogen from short durations of play outside the home in Kisumu is certain, due to the pervasiveness of pathogen contamination in these neighborhoods and frequent hand-to-soil and hand-to-mouth contacts. Multi-pathogen exposure was common for even the lowest risk behaviors; for example, the probability for exposure to 2 or more pathogens for one soil-hand-mouth contact during site-level play was 71%, although exposure to all pathogens was unlikely at even 10 contacts (< 35%). Contact with surface water was more dangerous, with only a few contacts resulting in exposure to all six pathogens. In reality the rate of different child behaviors likely vary between children and across populations of children, so the ranking of actual exposure risks associated with different behavioral, environmental, and spatial conditions should be evaluated based upon specific cultural settings. In the absence of quantitative data on child behavior in Kisumu public areas, we chose to keep contact frequency of behaviors at a constant rate to examine relative relationships between dose and diversity. Nonetheless, this data shows the threshold of safe contact between children and public domains in Kisumu for exposure to pathogens is low.

Another notable discovery was that exposure of the child to multiple public locations in their neighborhood, such as with free roaming child play, significantly increased pathogen doses and probability of multi-pathogen ingestion. Consideration of space as a determinant of environmental exposure to fecally-transmitted pathogens is rarely explicitly included as a function of exposure modeling, and when it is, spatial units tend to be defined using households or clusters of households as units of study. Our prior studies suggest that young children in urban, low-income settings–even those less than 24 months–are not immobile elements whose environmental exposures can be defined by these architectural boundaries.[34] While this study provides theoretical insight on potential child exposure conditions if children’s range of movement is considered the unit of study, very little is actually known about how much time children in low income settings spend in public areas and how far they roam. Even within a <25 m radius area, such as area just outside a child’s compound, our evidence of extreme covariance in pathogen concentrations and pathogen diversity in soil highlights the need for caution in estimating exposure risks from soil-hand-to-mouth and geophagy pathways with small sample sizes and with single organism microbial indicators. Accounting for microbial covariance in the environment during study design and modeling could improve reporting on model uncertainty and prevent conclusions from being biased by inadequate study design.

There were some pathogen-specific differences in exposure dose concentration curves. *Cryptosporidium spp*. was the most common pathogen in soil, at relatively stable concentrations across the neighborhood, which led to higher exposure doses. In this study we did not determine how many of these samples were *C*. *parvum* or *C*. *hominus* types, which are typically considered responsible for human infections. Although the public health importance of these exposures remains unclear, the 18S subunit gene indicator used in this study to detect Cryptosporidium was the same indicator used in a study of diarrhea in children less than two years of age in Bangladesh, which found *C*. *meleagridis* species are also common causes of child infection.[43] Pathogenic *E*. *coli* dose curves generally behaved similarly to each other, although soil appears to be a less common transmission pathway for EPEC. Human adenovirus and Giardia concentrations were highly varied between sites, compared to other pathogens, which indicates that child exposure to multiple public sites was an important determinant of greater doses.

This study has several important limitations. Concentrations of enteric viruses, bacteria, and protozoan pathogens were estimated by quantitative reverse transcription Polymerase Chain Reaction (qRT-PCR). While use of a multi-pathogen qRT-PCR process reduced methodological sources of variability in concentration estimates, and ergo exposure doses, it does not distinguish between viable (culturable), non-viable (live but non-culturable), and dead organisms. Pathogen exposure doses presented in this study may overestimate the number of infectious pathogens children ingest through contact with soil and surface water. There is a lack of information on how well qRT-PCR correlates with other approaches for quantifying pathogen environmental exposure, and with child disease outcomes in settings like Kisumu. PCR detection of microbial source tracking (MST) markers in Indian households was associated with diarrhea symptoms in one study.[22] However, MST markers may occur in the environment more frequently than infectious pathogens, and it is unclear whether MST presence is a reliable proxy for pathogen dose. Our model assumes pathogen concentrations follow a lognormal distribution; further the joint posterior distribution was largely reliant on the Tobit formulation and LLODs used to estimate the distribution of a high proportion of truncated values (non-detects). These modeling assumptions are difficult to verify due to the high proportion of non-detects. Because it is unlikely that very low doses result in meaningful exposures, we excluded doses below 1 DNA when estimating the probability of exposure to multiple pathogens. An additional limitation is our reliance on contact fate parameters from the extant literature. An important facet for future research would be to test the sensitivity of our conclusions to variations in these fate parameters.

Although validating health outcomes with exposure doses in Kisumu children was outside the scope of this study, our findings of pervasive environmental contamination and exposure to high doses of diverse pathogens are analogous to the county’s high prevalence of diarrhea in children under the age of five (18% of children experiencing diarrhea in a two-week prevalence).[44] Additionally, each of the pathogens common in soils and surface waters of Kisumu have been reported as leading causes of enteric infection in children in Kenya and elsewhere.[3] Further efforts should consider the importance of public exposure pathways in pediatric enteric infections. More information on dose-response in children in low income settings that accounts for potential interdependencies in the presence of pathogens would advance our understanding of exposure and infection outcomes.

The findings of probable pathogen exposure among young children who play in public areas provide one plausible explanation for why recent large scale trials of household WASH interventions have reported little [45] or no impact on child diarrhea [46] and why the global diarrhea disease burden remains high despite marked improvements in basic water and sanitation access in recent decades.[47] Child play outside the household is far more common than appreciated [34] and is likely to continue as long as families live in crowded conditions. Children playing unaccompanied or being cared for by older siblings may contribute to hazardous scenarios where children play in neighborhood areas and contact objects that are unsafe or unsanitary. Household WASH interventions do not typically address the topic of where children play, and do not install barriers between children and soil and water contaminated by the feces of one’s neighbors and domestic animals. Thus, while household WASH improvements can reduce child exposure and infection from some pathways, they may not sufficiently reduce exposure across all pathways to observe differences in diarrhea rates. To our knowledge, no household WASH trials have explicitly assessed whether children in intervention households have contact with public areas outside their compound. Thus, it remains unclear whether public exposures diminish the impact of improved household WASH conditions. Most of the recent large trials were conducted in rural areas where children’s probability of exposure to human feces in the neighborhood may be lower than for children in crowded urban Kisumu. [46] Yet, while human sanitation issues may vary across rural and urban settings, domestic animal feces is a universal environmental contamination issue across rural and urban settings.

In conclusion, our results suggest that exposure to public residential areas poses an important risk for enteric pathogen ingestion for children living in low-income settings with poor sanitary conditions, especially when those behaviors are frequent and of high volume, involve contact with surface water, and occur at multiple locations in the child’s neighborhood. Addressing these exposures will require broader WASH interventions targeting both child play behaviors and the environmental conditions in which play occurs. Future research should address the scarcity of information about spatial patterns of child behavior and the importance of public exposures in child infection outcomes.

## Supporting information

S1 Text Statistical analyses and additional details of the multivariate tobit model including the random effects for spatial dependence and Gibbs sampling algorithm. (DOCX)

S1 Fig 95% credible intervals (CI) for sample- and site-level correlation in estimated concentrations of enteric viruses, bacteria, and protozoans in soil and surface water in Kisumu, Kenya. (DOCX)

S2 Fig Mean pathogen doses with increased soil and surface water hand-to-mouth contact at site-restricted levels of spatial scale. (DOCX)

S3 Fig Mean pathogen doses with increased soil and surface water hand-to-mouth contact at neighborhood levels of spatial scale. (DOCX)

S4 Fig Mean pathogen doses with increased soil and surface water direct ingestion (geophagia and drinking water) at site-restricted levels of spatial scale. (DOCX)

S5 Fig Mean pathogen doses with increased soil and surface water direct ingestion (geophagia and drinking water) at neighborhood levels of spatial scale. (DOCX)

S6 Fig Dose distribution of Cryptosporidium ingested with increased frequency of soil and surface water contact at site-restricted versus neighborhood levels of spatial scale. (DOCX)

S7 Fig Dose distribution of Giardia ingested with increased frequency of soil and surface water contact at site-restricted versus neighborhood levels of spatial scale. (DOCX)

S8 Fig Dose distribution of human adenovirus 40/41 ingested with increased frequency of soil and surface water contact at site-restricted versus neighborhood levels of spatial scale. (DOCX)

S9 Fig Dose distribution of ETEC ingested with increased frequency of soil and surface water contact at site-restricted versus neighborhood levels of spatial scale. (DOCX)

S10 Fig Dose distribution of EPEC ingested with increased frequency of soil and surface water contact at site-restricted versus neighborhood levels of spatial scale. (DOCX)

S11 Fig Dose distribution of EAEC ingested with increased frequency of soil and surface water contact at site-restricted versus neighborhood levels of spatial scale. (DOCX)

S12 Fig Dose distribution of six enteric pathogens ingested with increased frequency of soil contact, site-level, for age groups: 6 to <12 month, 12 to <24 month, and 24 to <72 months of age. (DOCX)

S13 Fig Dose distribution of six enteric pathogens ingested with increased frequency of soil contact, neighborhood-level, for age groups: 6 to <12 month, 12 to <24 month, and 24 to <72 months of age. (DOCX)

S14 Fig Dose distribution of six enteric pathogens ingested with increased frequency of surface water contact, site-level, for age groups: 6 to <12 month, 12 to <24 month, and 24 to <72 months of age. (DOCX)

S15 Fig Dose distribution of six enteric pathogens ingested with increased frequency of surface water contact, neighborhood-level, for age groups: 6 to <12 month, 12 to <24 month, and 24 to <72 months of age. (DOCX)

S1 Table Summary of pathogens detected in environmental samples in three Kisumu neighborhoods and respective lower limits of detection. (DOCX)

S2 Table Detection frequencies for uncommon pathogens in soils and surface water from residential public areas of Kisumu. (DOCX)

S3 Table Reproducibility in detection of six enteric pathogens within a 25-meter radius area of 15 sites where multiple spatially-distinct soil and/or surface water samples were collected. (DOCX)

S4 Table Mean concentration of six enteric pathogens for 5 soil-hand mouth contacts, site-level, for age groups: 6 to <12, 12 to <24, and 24 to <72 months of age. (DOCX)

S5 Table Mean concentration of six enteric pathogens for 5 soil-hand mouth contacts, neighborhood-level, for age groups: 6 to <12, 12 to <24, and 24 to <72 months of age. (DOCX)

S6 Table Mean concentration of six enteric pathogens for 5 surface water-hand mouth contacts, site-level, for age groups: 6 to <12, 12 to <24, and 24 to <72 months of age. (DOCX)

S7 Table Mean concentration of six enteric pathogens for 5 surface water-hand mouth contacts, neighborhood-level, for age groups: 6 to <12, 12 to <24, and 24 to <72 months of age. (DOCX)

## References

[1] Fischer Walker CL, Perin J, Aryee MJ, Boschi-Pinto C, Black RE. Diarrhea incidence in low- and middle-income countries in 1990 and 2010: a systematic review. BMC Public Health. 2012;12:220 Epub 2012/03/23. 10.1186/1471-2458-12-220 22436130 PMC3323412

[2] Kotloff KL, Nataro JP, Blackwelder WC, Nasrin D, Farag TH, Panchalingam S. Burden and aetiology of diarrhoeal disease in infants and young children in developing countries (the Global Enteric Multicenter Study, GEMS): a prospective, case-control study. Lancet. 2013;382 10.1016/s0140-6736(13)60844-2 23680352

[3] Liu J, Platts-Mills JA, Juma J, Kabir F, Nkeze J, Okoi C, et al Use of quantitative molecular diagnostic methods to identify causes of diarrhoea in children: a reanalysis of the GEMS case-control study. Lancet. 2016;388(10051):1291–301. Epub 2016/09/28. 10.1016/S0140-6736(16)31529-X 27673470 PMC5471845

[4] The MAL-ED Study: A Multinational and Multidisciplinary Approach to Understand the Relationship Between Enteric Pathogens, Malnutrition, Gut Physiology, Physical Growth, Cognitive Development, and Immune Responses in Infants and Children Up to 2 Years of Age in Resource-Poor Environments. Clinical Infectious Diseases. 2014;59(suppl_4):S193–S206. 10.1093/cid/ciu653 25305287

[5] Platts-Mills JA, Babji S, Bodhidatta L, Gratz J, Haque R, Havt A. Pathogen-specific burdens of community diarrhoea in developing countries: a multisite birth cohort study (MAL-ED). Lancet Glob Health. 2015;3 10.1016/s2214-109x(15)00151-5 PMC7328884 26202075

[6] Shrivastava AK, Kumar S, Mohakud NK, Suar M, Sahu PS. Multiple etiologies of infectious diarrhea and concurrent infections in a pediatric outpatient-based screening study in Odisha, India. Gut Pathogens. 2017;9(1):16 10.1186/s13099-017-0166-0 28400860 PMC5387278

[7] Langendorf C, Hello S, Moumouni A, Gouali M, Mamaty AA, Grais RF. Enteric bacterial pathogens in children with diarrhea in Niger: diversity and antimicrobial resistance. PLoS ONE. 2015;10 10.1371/journal.pone.0120275 25799400 PMC4370739

[8] Bonkoungou IJO, Haukka K, Österblad M, Hakanen AJ, Traoré AS, Barro N. Bacterial and viral etiology of childhood diarrhea in Ouagadougou, Burkina Faso. BMC Pediatr. 2013;13 10.1186/1471-2431-13-36 23506294 PMC3616825

[9] Breurec S, Vanel N, Bata P, Chartier L, Farra A, Favennec L. Etiology and epidemiology of diarrhea in hospitalized children from low income country: a matched case–control study in Central African Republic. PLoS Negl Trop Dis. 2016;10 10.1371/journal.pntd.0004283 26731629 PMC4701495

[10] Platts-Mills JA, Babji S, Bodhidatta L, Gratz J, Haque R, Havt A, et al Pathogen-specific burdens of community diarrhoea in developing countries: a multisite birth cohort study (MAL-ED). Lancet Glob Health. 2015;3(9):e564–75. 10.1016/S2214-109X(15)00151-5 . 26202075 PMC7328884

[11] Kotloff KL, Nataro JP, Blackwelder WC, Nasrin D, Farag TH, Panchalingam S, et al Burden and aetiology of diarrhoeal disease in infants and young children in developing countries (the Global Enteric Multicenter Study, GEMS): a prospective, case-control study. Lancet. 2013;382(9888):209–22. Epub 2013/05/18. 10.1016/S0140-6736(13)60844-2 . 23680352

[12] Wagner EG, Lanoix JN. Excreta disposal for rural areas and small communities. Excreta Disposal for Rural Areas and Small Communities. 1958. 13581743

[13] Mattioli MC, Davis J, Boehm AB. Hand-to-mouth contacts result in greater ingestion of feces than dietary water consumption in Tanzania: a quantitative fecal exposure assessment model. Environmental science & technology. 2015;49(3):1912–20. 10.1021/es505555f . 25559008

[14] Julian TR, Pickering AJ. A Pilot Study on Integrating Videography and Environmental Microbial Sampling to Model Fecal Bacterial Exposures in Peri-Urban Tanzania. PLoS One. 2015;10(8):e0136158 Epub 2015/08/22. 10.1371/journal.pone.0136158 26295964 PMC4546663

[15] Labite H, Lunani I, van der Steen P, Vairavamoorthy K, Drechsel P, Lens P. Quantitative Microbial Risk Analysis to evaluate health effects of interventions in the urban water system of Accra, Ghana. J Water Health. 2010;8(3):417–30. Epub 2010/03/09. 10.2166/wh.2010.021 20375471

[16] Pickering AJ, Ercumen A, Arnold BF, Kwong LH, Parvez SM, Alam M, et al Fecal Indicator Bacteria along Multiple Environmental Transmission Pathways (Water, Hands, Food, Soil, Flies) and Subsequent Child Diarrhea in Rural Bangladesh. Environ Sci Technol. 2018 Epub 2018/06/15. 10.1021/acs.est.8b00928 . 29902374 PMC7705120

[17] Wang Y, Moe CL, Null C, Raj SJ, Baker KK, Robb KA, et al Multipathway Quantitative Assessment of Exposure to Fecal Contamination for Young Children in Low-Income Urban Environments in Accra, Ghana: The SaniPath Analytical Approach. Am J Trop Med Hyg. 2017;97(4):1009–19. Epub 2017/10/17. 10.4269/ajtmh.16-0408 29031283 PMC5637579

[18] Wang Y, Moe CL, Teunis PFM. Children Are Exposed to Fecal Contamination via Multiple Interconnected Pathways: A Network Model for Exposure Assessment. Risk Analysis. 2018 10.1111/risa.13146 30053314 PMC6282741

[19] Chigor VN, Sibanda T, Okoh AI. Assessment of the Risks for Human Health of Adenoviruses, Hepatitis A Virus, Rotaviruses and Enteroviruses in the Buffalo River and Three Source Water Dams in the Eastern Cape. Food and Environmental Virology. 2014;6(2):87–98. 10.1007/s12560-014-9138-4 24676673

[20] Crabtree KD, Gerba CP, Rose JB, Haas CN. Waterborne adenovirus: A risk assessment. Water Science and Technology. 1997;35(11):1–6. 10.1016/S0273-1223(97)00225-4.

[21] Englehardt JD, Swartout J. Predictive population dose-response assessment for Cryptosporidium parvum: infection endpoint. J Toxicol Environ Health A. 2004;67(8–10):651–66. 10.1080/15287390490428080 . 15192860

[22] Odagiri M, Schriewer A, Daniels ME, Wuertz S, Smith WA, Clasen T, et al Human fecal and pathogen exposure pathways in rural Indian villages and the effect of increased latrine coverage. Water Res. 2016;100:232–44. 10.1016/j.watres.2016.05.015 27192358 PMC4907306

[23] Pickering AJ, Julian TR, Marks SJ, Mattioli MC, Boehm AB, Schwab KJ, et al Fecal contamination and diarrheal pathogens on surfaces and in soils among Tanzanian households with and without improved sanitation. Environmental science & technology. 2012;46(11):5736–43. Epub 2012/05/02. 10.1021/es300022c . 22545817

[24] Baker KK, Senesac R, Sewell D, Sen Gupta A, Cumming O, Mumma J. Fecal Fingerprints of Enteric Pathogen Contamination in Public Environments of Kisumu, Kenya, Associated with Human Sanitation Conditions and Domestic Animals. Environmental Science & Technology. 2018;52(18):10263–74. 10.1021/acs.est.8b01528 30106283 PMC6557411

[25] Genthe B, Le Roux WJ, Schachtschneider K, Oberholster PJ, Aneck-Hahn NH, Chamier J. Health risk implications from simultaneous exposure to multiple environmental contaminants. Ecotoxicol Environ Saf. 2013;93:171–9. Epub 2013/05/15. 10.1016/j.ecoenv.2013.03.032 . 23669339

[26] Bauza V, Byrne DM, Trimmer JT, Lardizabal A, Atiim P, Asigbee MAK, et al Child soil ingestion in rural Ghana–frequency, caregiver perceptions, relationship with household floor material and associations with child diarrhoea. Tropical Medicine & International Health. 2018;23(5):558–69. 10.1111/tmi.13050 29537690

[27] Ngure FM, Humphrey JH, Mbuya MNN, Majo F, Mutasa K, Govha M, et al Formative Research on Hygiene Behaviors and Geophagy among Infants and Young Children and Implications of Exposure to Fecal Bacteria. The American Journal of Tropical Medicine and Hygiene. 2013;89(4):709–16. 10.4269/ajtmh.12-0568 24002485 PMC3795101

[28] AuYeung W, Canales RA, Beamer P, Ferguson AC, Leckie JO. Young children’s hand contact activities: An observational study via videotaping in primarily outdoor residential settings. J Expos Sci Environ Epidemiol. 2006;16(5):434–46. 10.1038/sj.jes.7500480 16552427

[29] Beamer P, Key ME, Ferguson AC, Canales RA, Auyeung W, Leckie JO. Quantified activity pattern data from 6-to-27-month-old farmworker children for use in exposure assessment. Environmental research. 2008;108(2):239–46. 10.1016/j.envres.2008.07.007 18723168 PMC2613792

[30] Ko S, Schaefer PD, Vicario CM, Binns HJ. Relationships of video assessments of touching and mouthing behaviors during outdoor play in urban residential yards to parental perceptions of child behaviors and blood lead levels. Journal Of Exposure Science And Environmental Epidemiology. 2006;17:47 10.1038/sj.jes.7500519 16941017

[31] Reed KJ, Jimenez M, Freeman NC, Lioy PJ. Quantification of children’s hand and mouthing activities through a videotaping methodology. Expo Anal Environ Epidemiol. 1999;9:513–20. 10.1038/sj.jea.7500047 10554153

[32] U.S. Environmental Protection Agency (EPA). Exposure Factors Handbook: 2011 Edition. Washington, DC: National Center for Environmental Assessment; 2011.

[33] Gretsch SR, Ampofo JA, Baker KK, Clennon J, Null CA, Peprah D, et al Quantification of exposure to fecal contamination in open drains in four neighborhoods in Accra, Ghana. J Water Health. 2016;14(2):255–66. 10.2166/wh.2015.138 . 27105411

[34] Medgyesi DN, Brogan JM, Sewell DK, Creve-Coeur JP, Kwong LH, Baker KK. Where Children Play: Young child exposure to environmental hazards during play in public areas in a transitioning internally displaced persons community in Haiti. International Journal of Environmental Research and Public Health. 2018;15(8):1682 Epub August 3, 2018. 10.3390/ijerph15081682. PMC6122025 30081490

[35] Xue J, Zartarian V, Moya J, Freeman N, Beamer P, Black K, et al A meta-analysis of children’s hand-to-mouth frequency data for estimating nondietary ingestion exposure. Risk Analysis: An International Journal. 2007;27(2):411–20. 10.1111/j.1539-6924.2007.00893.x 17511707

[36] Kwong LH, Ercumen A, Pickering AJ, Unicomb L, Davis J, Luby SP. Hand- and Object-Mouthing of Rural Bangladeshi Children 3–18 Months Old. Int J Environ Res Public Health. 2016;13(6). 10.3390/ijerph13060563 . 27271651 PMC4924020

[37] Medgyesi DN, Brogan JM, Sewell DK, Creve-Coeur JP, Kwong LH, Baker KK. Where Children Play: Young child exposure to environmental hazards during play in public areas in a transitioning internally displaced persons community in Haiti. International Journal of Environmental Research and Public Health. 2018;15(8):1646. 10.3390/ijerph15081646 PMC6122025 30081490

[38] US EPA. Methods for Assessing Exposure to Chemical Substances: Volume 7 Washington, D.C: US Environmental Protection Agency; 1987.

[39] Finley BL, Scott PK, Mayhall DA. Development of a Standard Soil‐to‐Skin Adherence Probability Density Function for Use in Monte Carlo Analyses of Dermal Exposure. Risk Analysis. 1994;14(4):555–69. 10.1111/j.1539-6924.1994.tb00270.x 7972958

[40] AuYeung W, Canales RA, Leckie JO. The fraction of total hand surface area involved in young children’s outdoor hand-to-object contacts. Environmental Research. 2008;108(3):294–9. 10.1016/j.envres.2008.07.010. 18760778

[41] Rusin P, Maxwell S, Gerba C. Comparative surface-to-hand and fingertip-to-mouth transfer efficiency of gram-positive bacteria, gram-negative bacteria, and phage. Journal of Applied Microbiology. 2002;93(4):585–92. 10.1046/j.1365-2672.2002.01734.x 12234341

[42] AuYeung W, Canales RA, Beamer P, Ferguson AC, Leckie JO. Young Children’s Mouthing Behavior: An Observational Study via Videotaping in a Primarily Outdoor Residential Setting. Journal of Children’s Health. 2005;2(3–4):271–95. 10.3109/15417060490960215 16552427

[43] Steiner KL, Ahmed S, Gilchrist CA, Burkey C, Cook H, Ma JZ, et al Species of Cryptosporidia Causing Subclinical Infection Associated with Growth Faltering in Rural and Urban Bangladesh- a Birth Cohort Study. Clin Infect Dis. 2018 Epub 2018/06/14. 10.1093/cid/ciy310 PMC6186860 29897482

[44] Kenya National Bureau of Statistics. Kenya—Multiple Indicator Cluster Survey 2011, Nyanza Province 2013. http://microdata.worldbank.org/index.php/catalog/2660.

[45] Luby SP, Rahman M, Arnold BF, Unicomb L, Ashraf S, Winch PJ, et al Effects of water quality, sanitation, handwashing, and nutritional interventions on diarrhoea and child growth in rural Bangladesh: a cluster randomised controlled trial. The Lancet Global Health. 2018;6(3):e302–e15. 10.1016/S2214-109X(17)30490-4 29396217 PMC5809718

[46] Null C, Stewart CP, Pickering AJ, Dentz HN, Arnold BF, Arnold CD, et al Effects of water quality, sanitation, handwashing, and nutritional interventions on diarrhoea and child growth in rural Kenya: a cluster-randomised controlled trial. The Lancet Global Health. 2018;6(3):e316–e29. 10.1016/S2214-109X(18)30005-6 29396219 PMC5809717

[47] Walker CLF, Rudan I, Liu L, Nair H, Theodoratou E, Bhutta ZA. Global burden of childhood diarrhoea and pneumonia. Lancet. 2013;381. 10.1016/S0140-6736(13)60222-6 PMC7159282 23582727

