# Supplementary material for: The landscape of enteric pathogen exposure of young children in public domains of low-income, urban Kenya: The influence of exposure pathway and spatial range of play on multi-pathogen exposure risks
Source: PLoS Negl Trop Dis. 2019 Mar 27;13(3):e0007292. doi: 10.1371/journal.pntd.0007292 (PMC6453472; doi:10.1371/journal.pntd.0007292)
Supplement: S1 Text — (DOCX) [file pntd.0007292.s001.docx]

**S1 Text.**

Statistical Analyses.

Without loss of generality, we may assume that the lower limit of detection (LLOD) for each pathogen is zero. In practice, this implies that each of the log pathogen concentrations was shifted to the left by its corresponding LLOD; to readjust the estimation output, we simply added the LLOD back to the mean vector. The multivariate random effects tobit model we utilized can be written as


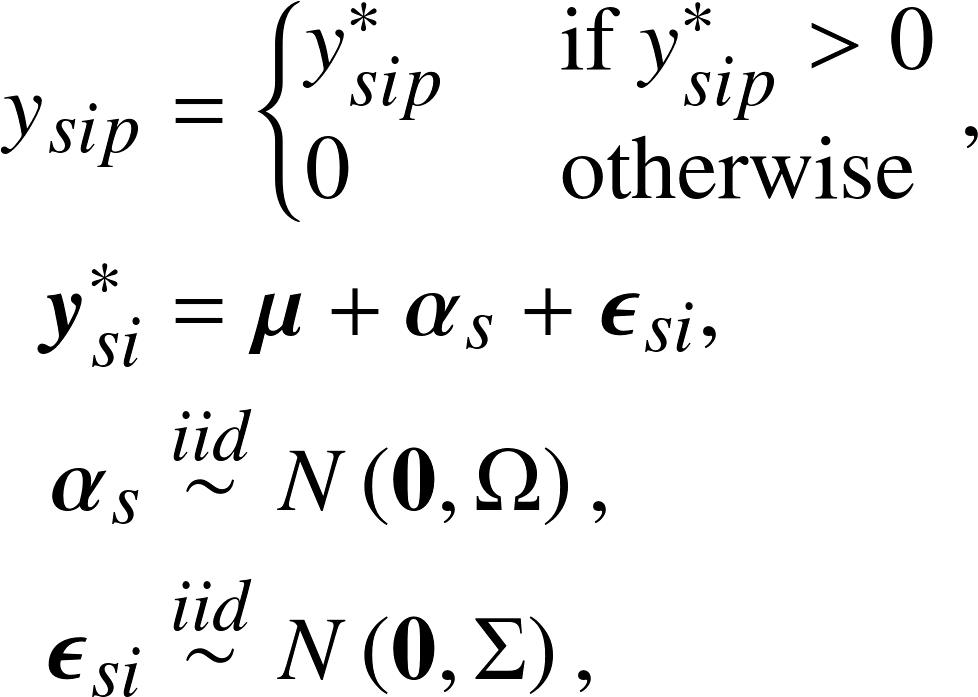


for
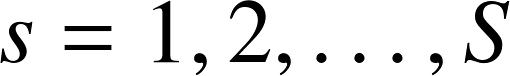
,
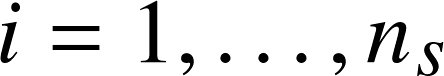
, and
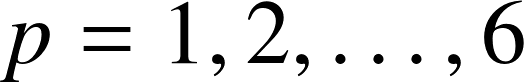
, where
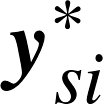
 is the 6-dimensional vector of the untruncated pathogen concentrations for the
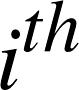
 replicate at sample site *s*, *S* is the total number of sites,
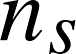
 is the number of replicate samples at site *s*,
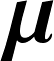
 is the 6-dimensional vector of pathogen concentration means,
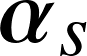
 is the 6-dimensional random effect capturing the correlation structure between replicate samples within each site,
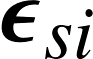
 is the 6-dimensional residual variation within each sample, and
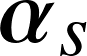
 and
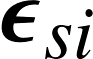
 are independent for all *s* and *i*,
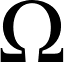
 is the 6x6 covariance matrix for the random effects, and
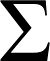
 is the 6x6 covariance matrix for the residuals. The number of parameters in this model is 48 (
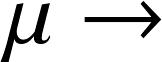
 6,
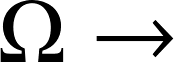
 21,
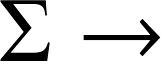
 21 = 48).

There are two sources of dependency in the data accounted for in this model- the inherent noise in the system as captured by
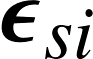
 and the spatial correlation captured by
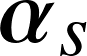
 . Hence the covariance matrix corresponding to the untruncated pathogen concentrations for a sample is given by
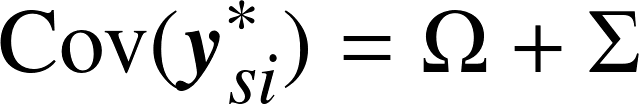
, and the covariance matrix corresponding to the untruncated pathogen concentration between two different samples taken at the same site is given by
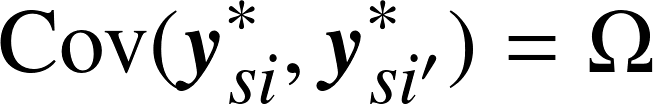
 . Hence the correlation for the *p*^th^ pathogen between two samples at the same site can be found via the intra-class correlation (ICC):
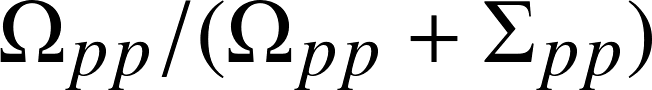
, and the total variance of the *p*^th^ pathogen log concentration is
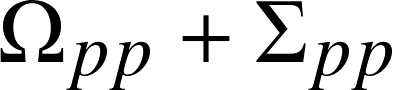
. Of course, samples taken at two different sites are independent.

In the main manuscript, the parameters of the multivariate random effects tobit model was generally notated as
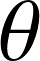
. Note that here
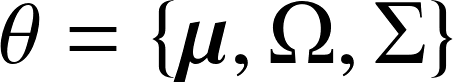
 .

Draws from the posterior distribution of
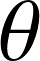
 given the data were obtained through a Gibbs sampler. The priors are given as


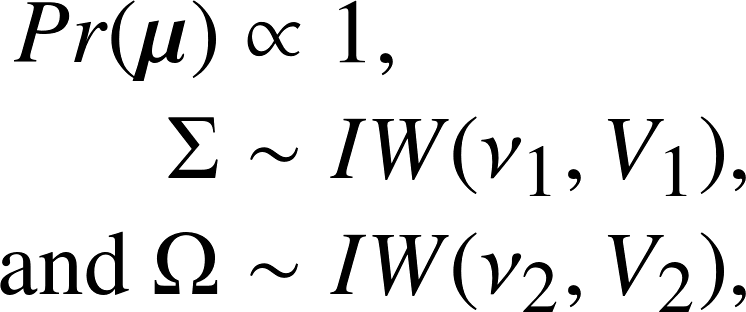


where
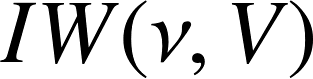
 represents the inverse Wishart distribution with
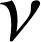
 degrees of freedom and scale matrix *V*. The full conditionals are provided below.


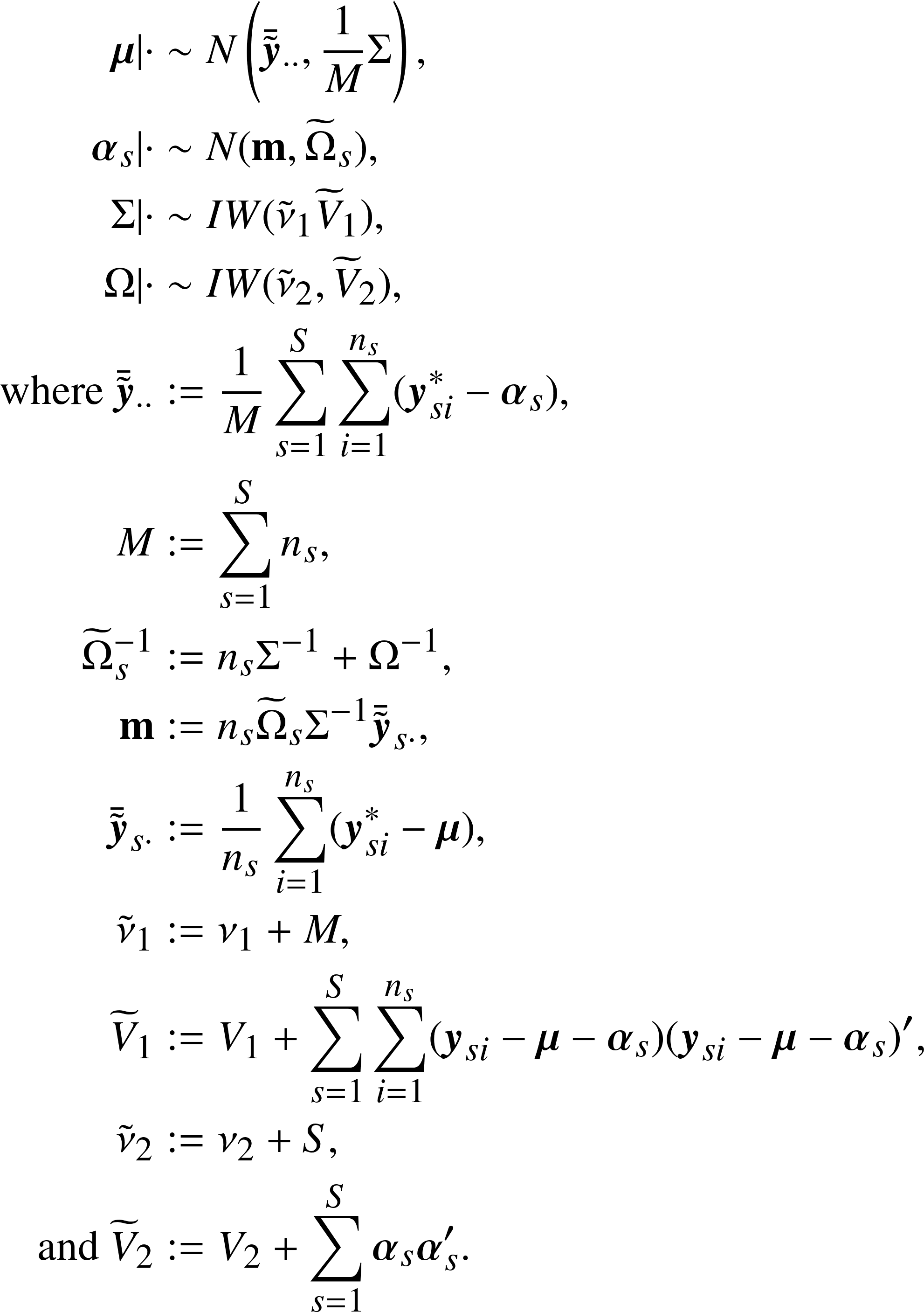


To draw from the full conditional distribution of
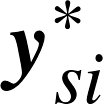
 , we first partition
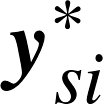
 into two parts: (1)
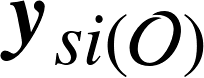
 defined to be the elements (i.e., pathogens) of
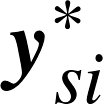
 that are in
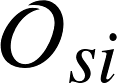
 , where
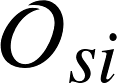
 is the set of pathogens in
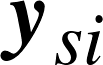
 observed above the LLOD, and (2)
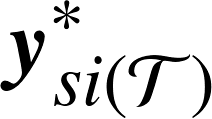
 defined to be the elements of
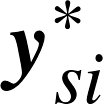
 that are in
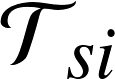
 , where
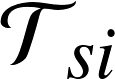
 is the set of pathogens in
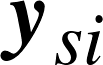
 which were truncated due to falling below the LLOD. Clearly
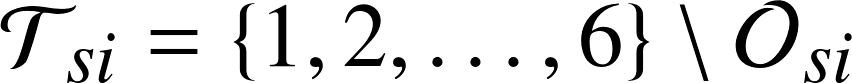
 . If
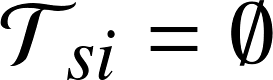
, then no draws must be made as there are no unknowns, otherwise we must sequentially make draws from a truncated normal distribution according to the truncated elements. That is, we decompose the full conditional distribution in the following way:


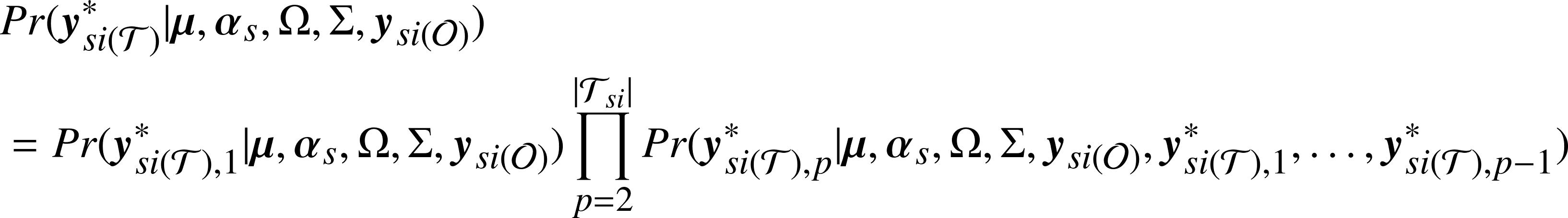


Each of these distributions on the right hand side of the equation are univariate truncated normal over the range
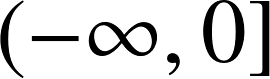
 . We begin by drawing the first truncated, i.e., unobserved, pathogen in
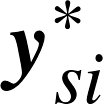
 from
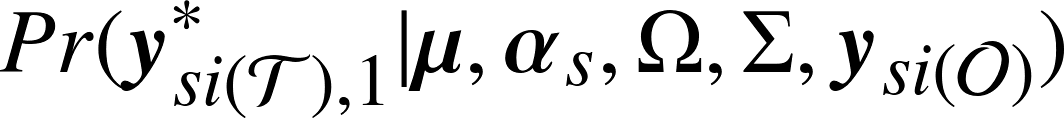
 , and then for
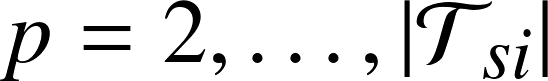
 draw the remaining unobserved pathogens in
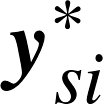
 from
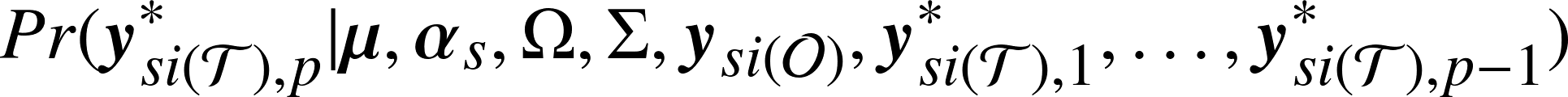
. This iterative procedure is overwhelmingly more computationally efficient than trying to draw directly from the full conditional distribution
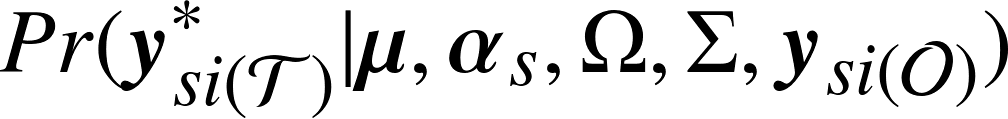
 using, e.g., rejection sampling.

References

Baker KK, Senesac R, Sewell D, Sen Gupta A, Cumming O, Mumma J. Fecal Fingerprints of Enteric Pathogen Contamination in Public Environments of Kisumu, Kenya, Associated with Human Sanitation Conditions and Domestic Animals. Environmental Science & Technology. 2018;52(18):10263-74. doi: 10.1021/acs.est.8b01528.
