# Supplementary material for: The landscape of enteric pathogen exposure of young children in public domains of low-income, urban Kenya: The influence of exposure pathway and spatial range of play on multi-pathogen exposure risks
Source: PLoS Negl Trop Dis. 2019 Mar 27;13(3):e0007292. doi: 10.1371/journal.pntd.0007292 (PMC6453472; doi:10.1371/journal.pntd.0007292)
Supplement: S1 Fig — (DOCX) [file pntd.0007292.s002.docx]

**S1 Fig.** The 95% credible intervals (CI) for sample- (A-D) and site-level (E-H) correlation in concentration of enteric viruses, bacteria, and protozoans in soil (left two columns) and surface water (right two columns) in Kisumu, Kenya. Negative and positive correlation shown in the lower left quadrants of each grid are reflected in orange-red and blue circles, respectively, in the upper right quadrants of each grid. The highest rho indicated by the darkest color and narrowest shapes. **
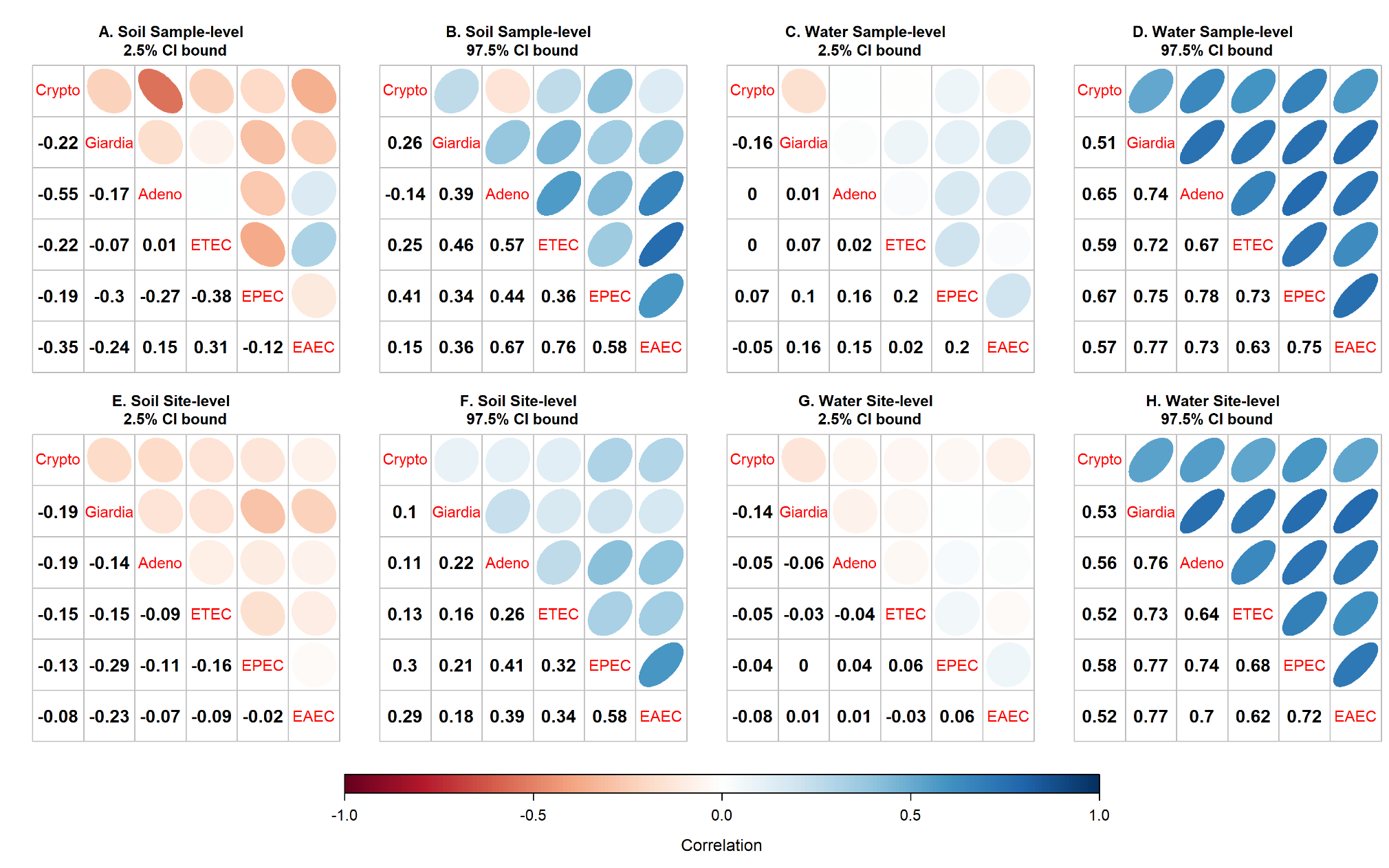
**
