# Supplementary material for: The landscape of enteric pathogen exposure of young children in public domains of low-income, urban Kenya: The influence of exposure pathway and spatial range of play on multi-pathogen exposure risks
Source: PLoS Negl Trop Dis. 2019 Mar 27;13(3):e0007292. doi: 10.1371/journal.pntd.0007292 (PMC6453472; doi:10.1371/journal.pntd.0007292)
Supplement: S2 Fig — (DOCX) [file pntd.0007292.s003.docx]

**S2 Fig.** Mean pathogen doses with increased soil and surface water hand-to-mouth contact at site-restricted levels of spatial scale.**
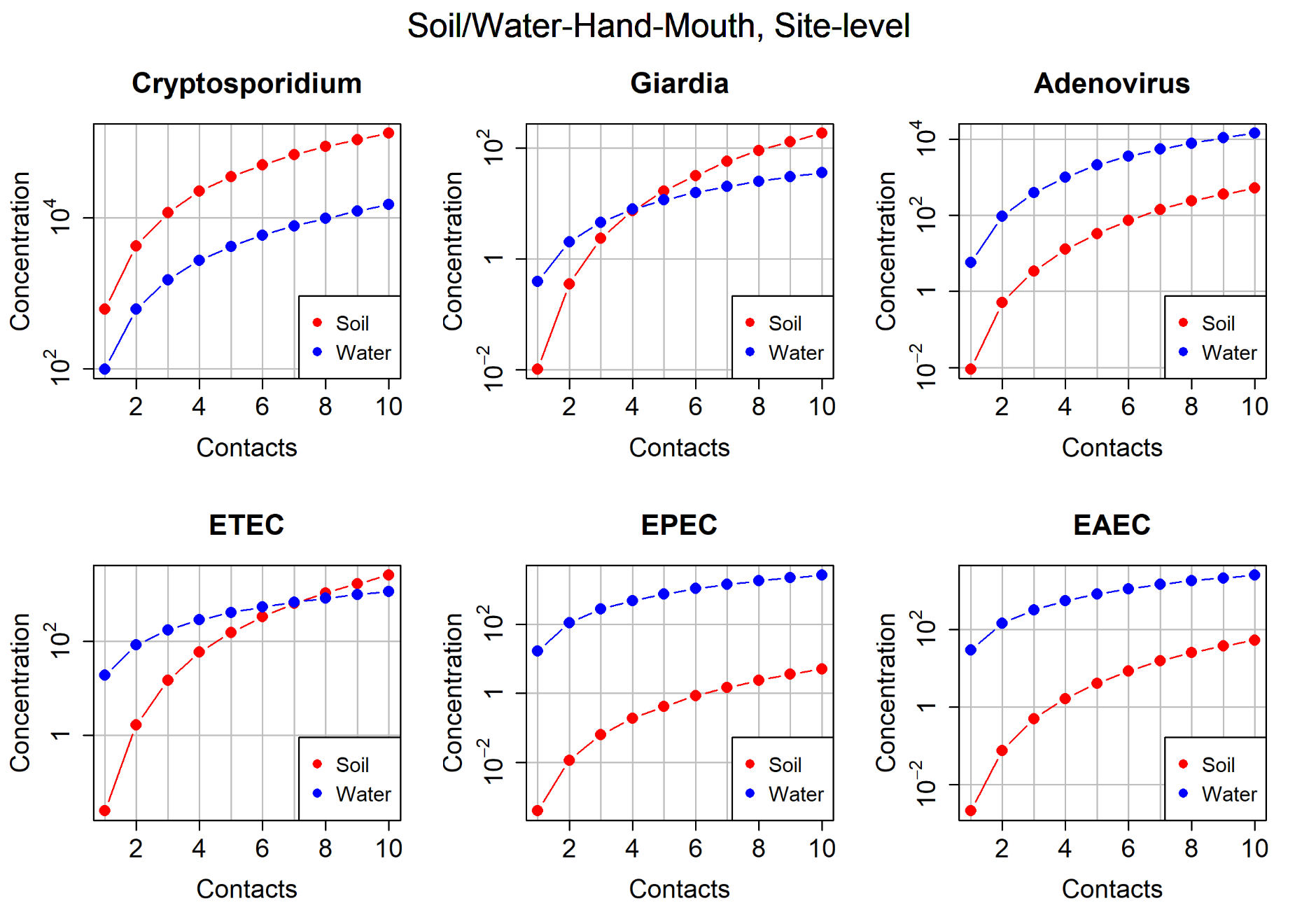
**
