# Supplementary material for: The landscape of enteric pathogen exposure of young children in public domains of low-income, urban Kenya: The influence of exposure pathway and spatial range of play on multi-pathogen exposure risks
Source: PLoS Negl Trop Dis. 2019 Mar 27;13(3):e0007292. doi: 10.1371/journal.pntd.0007292 (PMC6453472; doi:10.1371/journal.pntd.0007292)
Supplement: S5 Fig — (DOCX) [file pntd.0007292.s006.docx]

**S5 Fig.** Mean pathogen doses with increased soil and surface water direct ingestion (geophagia and drinking water) at neighborhood levels of spatial scale.**
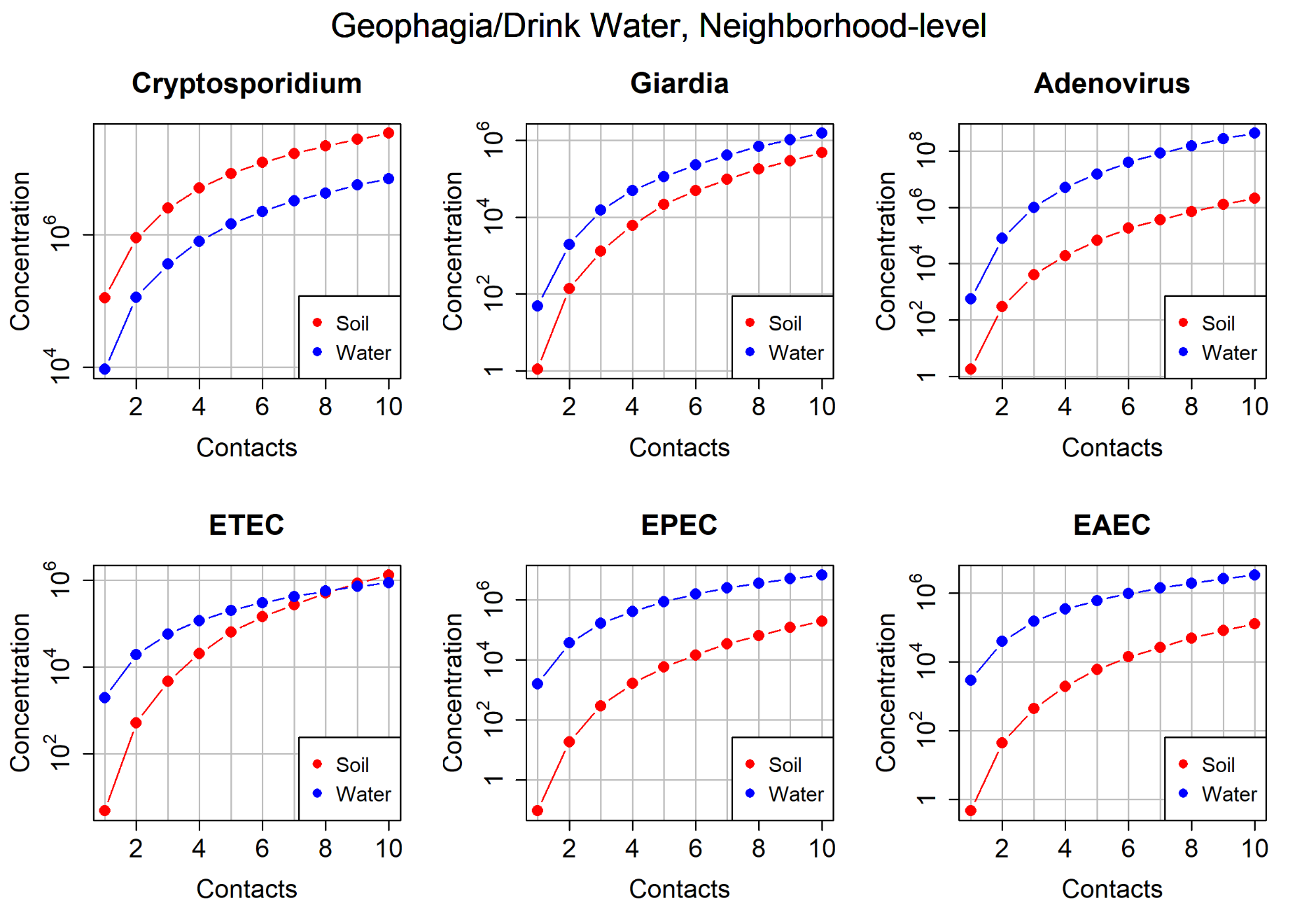
**
