# Supplementary material for: The landscape of enteric pathogen exposure of young children in public domains of low-income, urban Kenya: The influence of exposure pathway and spatial range of play on multi-pathogen exposure risks
Source: PLoS Negl Trop Dis. 2019 Mar 27;13(3):e0007292. doi: 10.1371/journal.pntd.0007292 (PMC6453472; doi:10.1371/journal.pntd.0007292)

**S9 Fig.** Dose distribution (mean and 25^th^ and 75^th^ quantiles) of ETEC ingested with increased frequency of soil and surface water contact at site-restricted versus neighborhood levels of spatial scale.


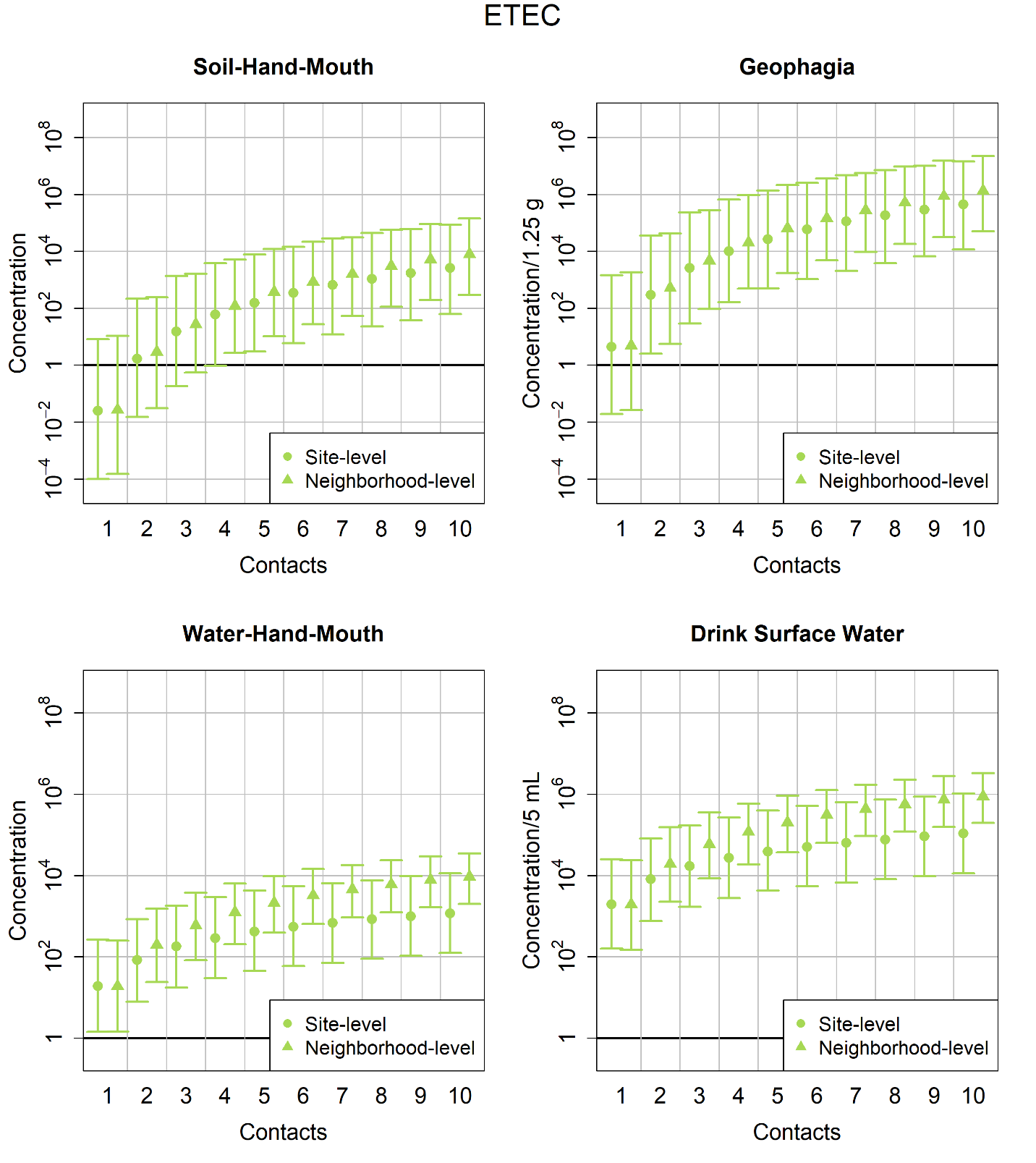

Supplement: S9 Fig — (DOCX) [file pntd.0007292.s010.docx]
