# Supplementary material for: The landscape of enteric pathogen exposure of young children in public domains of low-income, urban Kenya: The influence of exposure pathway and spatial range of play on multi-pathogen exposure risks
Source: PLoS Negl Trop Dis. 2019 Mar 27;13(3):e0007292. doi: 10.1371/journal.pntd.0007292 (PMC6453472; doi:10.1371/journal.pntd.0007292)
Supplement: S14 Fig — (DOCX) [file pntd.0007292.s015.docx]

**S14 Fig.** Dose distribution of six enteric pathogens ingested with increased frequency of surface water contact, site-level, for age groups: 6 to <12 month, 12 to <24 month, and 24 to <72 months of age.
