# Supplementary material for: The landscape of enteric pathogen exposure of young children in public domains of low-income, urban Kenya: The influence of exposure pathway and spatial range of play on multi-pathogen exposure risks
Source: PLoS Negl Trop Dis. 2019 Mar 27;13(3):e0007292. doi: 10.1371/journal.pntd.0007292 (PMC6453472; doi:10.1371/journal.pntd.0007292)
Supplement: S1 Table — (DOCX) [file pntd.0007292.s017.docx]

**S1 Table.** Mean concentration, standard deviation (SD), and lower limits of detection (LLOD) of enteric viruses, bacteria, and protozoans per gram of soil and milliliter (mL) of surface water in pathogen-positive samples from peri-domestic areas of three Kisumu neighborhoods.

| **Pathogen type** | **SOIL, n=125** | | | **WATER, n=34** | | |
| --- | --- | --- | --- | --- | --- | --- |
|  | Positive (%) | Geometric Mean/gram** (SD) | LLOD ^d^/  gram | Positive (%) | Geometric Mean/mL** (SD) | LLOD ^d^/ mL |
| *Cryptosporidium spp.* | 94  (75) | 5.49x10^5^  (3.85x10^0^) | 1.9x10^3^ | 23  (68) | 1.79x10^4^  (7.9x10^0^) | 9.7x10^1^ |
| *Giardia lamblia* | 19  (15) | 2.53x10^5^  (3.36x10^1^) | 9.5x10^2^ | 13  (38) | 8.02x10^3^  (2.3x10^0^) | 4.8x10^1^ |
| human adenovirus 40/41 | 11  (9) | 5.87x10^7^  (2.77x10^1^) | 2.6x10^4^ | 13  (38) | 4.79x10^5^  (2.83x10^1^) | 1.3x10^3^ |
| ^a^ ETEC *estA*/eltB* | 23  (18) | 7.51x10^5^  (5.08x10^0^) | 1.6x10^3^ | 21  (62) | 3.45x10^3^  (6.2x10^0^) | 8.0x10^1^ |
| ^b^ EPEC *bfpA*/eaeA* | 13  (10) | 1.32x10^6^  (7.42x10^0^) | 4.3x10^3^ | 17  (50) | 1.76x10^4^  (7.1x10^0^) | 2.1x10^2^ |
| ^c^ EAEC *aatA*/aaiC* | 16  (13) | 2.68x10^5^  (8.61x10^0^) | 3.2x10^3^ | 20  (59) | 8.16x10^3^  (1.02x10^1^) | 1.6x10^2^ |

^a^ Enterotoxigenic *E. coli* (ETEC)

^b^ Enteropathogenic *E. coli* (EPEC)

^c^ Enteroaggregative *E. coli* (EAEC)

^d^ Lower limit of detection (LLOD) described elsewhere.([1](#_ENREF_1))
