# Supplementary material for: The landscape of enteric pathogen exposure of young children in public domains of low-income, urban Kenya: The influence of exposure pathway and spatial range of play on multi-pathogen exposure risks
Source: PLoS Negl Trop Dis. 2019 Mar 27;13(3):e0007292. doi: 10.1371/journal.pntd.0007292 (PMC6453472; doi:10.1371/journal.pntd.0007292)
Supplement: S2 Table — (DOCX) [file pntd.0007292.s018.docx]

**S2 Table**. Detection frequencies for uncommon pathogens in soils and surface water from residential public areas of Kisumu.

| **Pathogen type** | **SOIL, n=125** | **WATER, n=34** |
| --- | --- | --- |
|  | Positive (%) | Positive (%) |
| Sapovirus | 0 | 4 (12) |
| Rotavirus | 0 | 1 (3) |
| Norovirus GII | 0 | 2 (6) |
| Astrovirus | 1 (1) | 1 (3) |
| *C. difficile* | 0 | 0 |
| *C. jejuni* | 5 (4) | 5 (15) |
| *V. cholera* | 4 (3) | 11 (32) |
| *Shigella/EIEC* | 1 (1) | 3 (9) |
| *Salmonella* | 2 (2) | 0 |
| Ascaris | 2 (2) | 0 |
| E. histolytica | 1 (1) | 0 |
| Trichuris | 2 (2) | 0 |
