# Supplementary material for: The landscape of enteric pathogen exposure of young children in public domains of low-income, urban Kenya: The influence of exposure pathway and spatial range of play on multi-pathogen exposure risks
Source: PLoS Negl Trop Dis. 2019 Mar 27;13(3):e0007292. doi: 10.1371/journal.pntd.0007292 (PMC6453472; doi:10.1371/journal.pntd.0007292)
Supplement: S3 Table — (DOCX) [file pntd.0007292.s019.docx]

**S3 Table**. Reproducibility in detection of six enteric pathogens within a 25-meter radius area of 15 sites where multiple spatially-distinct soil and/or surface water samples were collected.

|  | **Not detected (%)** | **Detected once (%)** | **Detected in multiple replicates (%)** | **Detected in all replicates (%)** |
| --- | --- | --- | --- | --- |
| Cryptosporidium spp. | 0 | 0 | 12 (80) | 3 (20) |
| Giardia lamblia | 9 (60) | 4 (27) | 2 (13) | 0 |
| human adenovirus 40/41 | 8 (53) | 5 (33) | 1 (7) | 1 (7) |
| ETEC | 8 (53) | 3 (20) | 4 (27) | 0 |
| EPEC | 8 (53) | 5 (33) | 2 (13) | 0 |
| EAEC | 6 (40) | 5 (33) | 4 (27) | 0 |

**Summary:** Overall, reproducibility in pathogen detection within a 25-meter radius was low, with repeat detection of the same type of pathogen for most pathogens occurring approximately one quarter to one-half of pathogen-positive sites. The exception was Cryptosporidium, which was detected at all 15 multi-sampling sites and thus had high rates of repeat detection at fine-scale levels of sampling. Notably, resampling within a site was designed to compare variance in pathogen detection at small-scale distances across the environment, rather than as a retest of environmental or methodological variability in detection at a specific set of GPS coordinates. Samples could have been as much as 50 meters apart, if on opposite ends of the 25 m radius site. These results are evidence of spatial heterogeneity in pathogen contamination of the environment.
