# Supplementary material for: The landscape of enteric pathogen exposure of young children in public domains of low-income, urban Kenya: The influence of exposure pathway and spatial range of play on multi-pathogen exposure risks
Source: PLoS Negl Trop Dis. 2019 Mar 27;13(3):e0007292. doi: 10.1371/journal.pntd.0007292 (PMC6453472; doi:10.1371/journal.pntd.0007292)
Supplement: S4 Table — (DOCX) [file pntd.0007292.s020.docx]

**S4 Table**. Mean concentration of six enteric pathogens for 5 soil-hand mouth contacts, site-level, for age groups: 6 to <12, 12 to <24, and 24 to <72 months of age.

|  | 6 to <12 months | 12 to <24 months | 24 to <72 months |
| --- | --- | --- | --- |
| Crypto | 2.59E+04 | 3.23E+04 | 3.72E+04 |
| Giardia | 1.26E+01 | 1.57E+01 | 1.81E+01 |
| Adeno | 2.43E+01 | 3.03E+01 | 3.50E+01 |
| ETEC | 1.15E+02 | 1.44E+02 | 1.66E+02 |
| EPEC | 3.05E-01 | 3.81E-01 | 4.39E-01 |
| EAEC | 3.00E+00 | 3.74E+00 | 4.32E+00 |
