# Supplementary material for: The landscape of enteric pathogen exposure of young children in public domains of low-income, urban Kenya: The influence of exposure pathway and spatial range of play on multi-pathogen exposure risks
Source: PLoS Negl Trop Dis. 2019 Mar 27;13(3):e0007292. doi: 10.1371/journal.pntd.0007292 (PMC6453472; doi:10.1371/journal.pntd.0007292)
Supplement: S5 Table — (DOCX) [file pntd.0007292.s021.docx]

**S5 Table**. Mean concentration of six enteric pathogens for 5 soil-hand mouth contacts, neighborhood-level, for age groups: 6 to <12, 12 to <24, and 24 to <72 months of age.

|  | 6 to <12 months | 12 to <24 months | 24 to <72 months |
| --- | --- | --- | --- |
| Crypto | 3.69E+04 | 4.61E+04 | 5.31E+04 |
| Giardia | 9.27E+01 | 1.16E+02 | 1.33E+02 |
| Adeno | 2.84E+02 | 3.54E+02 | 4.08E+02 |
| ETEC | 2.76E+02 | 3.44E+02 | 3.97E+02 |
| EPEC | 2.43E+01 | 3.03E+01 | 3.50E+01 |
| EAEC | 2.54E+01 | 3.16E+01 | 3.65E+01 |
