# Supplementary material for: The landscape of enteric pathogen exposure of young children in public domains of low-income, urban Kenya: The influence of exposure pathway and spatial range of play on multi-pathogen exposure risks
Source: PLoS Negl Trop Dis. 2019 Mar 27;13(3):e0007292. doi: 10.1371/journal.pntd.0007292 (PMC6453472; doi:10.1371/journal.pntd.0007292)
Supplement: S6 Table — (DOCX) [file pntd.0007292.s022.docx]

**S6 Table**. Mean concentration of six enteric pathogens for 5 surface water-hand mouth contacts, site-level, for age groups: 6 to <12, 12 to <24, and 24 to <72 months of age.

|  | 6 to <12 months | 12 to <24 months | 24 to <72 months |
| --- | --- | --- | --- |
| Crypto | 3.09E+03 | 3.85E+03 | 4.44E+03 |
| Giardia | 8.65E+00 | 1.08E+01 | 1.24E+01 |
| Adeno | 1.54E+03 | 1.93E+03 | 2.22E+03 |
| ETEC | 3.07E+02 | 3.83E+02 | 4.42E+02 |
| EPEC | 5.48E+02 | 6.83E+02 | 7.88E+02 |
| EAEC | 6.02E+02 | 7.51E+02 | 8.66E+02 |
