# Supplementary material for: The landscape of enteric pathogen exposure of young children in public domains of low-income, urban Kenya: The influence of exposure pathway and spatial range of play on multi-pathogen exposure risks
Source: PLoS Negl Trop Dis. 2019 Mar 27;13(3):e0007292. doi: 10.1371/journal.pntd.0007292 (PMC6453472; doi:10.1371/journal.pntd.0007292)
Supplement: S7 Table — (DOCX) [file pntd.0007292.s023.docx]

**S7 Table**. Mean concentration of six enteric pathogens for 5 surface water-hand mouth contacts, neighborhood-level, for age groups: 6 to <12, 12 to <24, and 24 to <72 months of age.

|  | 6 to <12 months | 12 to <24 months | 24 to <72 months |
| --- | --- | --- | --- |
| Crypto | 1.11E+04 | 1.38E+04 | 1.59E+04 |
| Giardia | 8.63E+02 | 1.08E+03 | 1.24E+03 |
| Adeno | 1.14E+05 | 1.42E+05 | 1.64E+05 |
| ETEC | 1.56E+03 | 1.95E+03 | 2.25E+03 |
| EPEC | 6.73E+03 | 8.39E+03 | 9.68E+03 |
| EAEC | 4.65E+03 | 5.79E+03 | 6.68E+03 |
